# 4-Amino-1,2,4-triazole-3-thione as a Promising Scaffold for the Inhibition of Serine and Metallo-*β*-Lactamases

**DOI:** 10.3390/ph13030052

**Published:** 2020-03-24

**Authors:** Pasquale Linciano, Eleonora Gianquinto, Martina Montanari, Lorenzo Maso, Pierangelo Bellio, Esmeralda Cebrián-Sastre, Giuseppe Celenza, Jesús Blázquez, Laura Cendron, Francesca Spyrakis, Donatella Tondi

**Affiliations:** 1 Department of Life Sciences, University of Modena and Reggio Emilia, Via G. Campi 103, 41125 Modena, Italy; p.linciano@unimore.it (P.L.); 186334@studenti.unimore.it (M.M.); 2 Department of Drug Science and Technology, University of Turin, Via P. Giuria 9, 10125 Turin, Italy; eleonora.gianquinto@unito.it; 3 Department of Biology, University of Padua, Viale G. Colombo 3, 35121 Padua, Italy; lorenzo.maso@unipd.it (L.M.); laura.cendron@unipd.it (L.C.); 4 Department of Biotechnological and Applied Clinical Sciences, University of L’Aquila, via Vetoio 1, 67100 L’Aquila, Italy; pierangelo.bellio@univaq.it (P.B.); giuseppe.celenza@univaq.it (G.C.); 5 National Center of Biotechnology-CSIC, Calle Darwin 3, 28049 Madrid, Spain; esmeralda.cebrian@cnb.csic.es (E.C.-S.); blazquez@cnb.csic.es (J.B.)

**Keywords:** 4-amino-1,2,4-triazole-3-thione, bacterial resistance, structure-based drug design, non-covalent inhibition, thione/thiol tautomerism, broad-spectrum activity

## Abstract

The emergence of bacteria that co-express serine- and metallo- carbapenemases is a threat to the efficacy of the available *β*-lactam antibiotic armamentarium. The 4-amino-1,2,4-triazole-3-thione scaffold has been selected as the starting chemical moiety in the design of a small library of *β*-Lactamase inhibitors (BLIs) with extended activity profiles. The synthesised compounds have been validated in vitro against class A serine *β−*Lactamase (SBLs) KPC-2 and class B1 metallo *β−*Lactamases (MBLs) VIM-1 and IMP-1. Of the synthesised derivatives, four compounds showed cross-class micromolar inhibition potency and therefore underwent *in silico* analyses to elucidate their binding mode within the catalytic pockets of serine- and metallo-BLs. Moreover, several members of the synthesised library have been evaluated, in combination with meropenem (MEM), against clinical strains that overexpress BLs for their ability to synergise carbapenems.

## 1. Introduction

The spread of multidrug-resistant (MDR) Gram-negative bacteria is a recognised public-health issue, compromising the efficacious treatment of bacterial infections, and becoming a leading cause of death worldwide [1]. Although there has recently been a resurgence in antibiotic drug discovery and new targets, mechanisms and molecular entities have been addressed [2,3,4], the need for continuous investment in antibiotic-drug development is necessary. At present, *β*-lactams remain the most commonly used antibiotics in bacterial infections worldwide. However, their misuse in humans and animals exposes bacteria to a massive pressure, leading to the selection of MDR microorganisms [5]. Among the several mechanisms that bacteria adopt to inactivate *β*-lactam antibiotics, the over-expression of the hydrolytic enzymes *β*-lactamases (BLs) remains the most relevant [6,7].

According to Ambler’s classification, *β*-lactamases are divided into four classes, A, B, C and D [8]. Classes A, C and D are serine-based enzymes (SBLs), whereas class B includes metallo-*β*-lactamases (MBLs) [6]. KPC-2 (*Klebsiella pneumoniae* carbapenemase-2) is the most prevalent SBL and has spread around the world since its discovery, only a few years ago, becoming endemic in some countries [9]. The clinical relevance of KPC-2 and its several variants relies on its capability to hydrolyse a broad variety of *β*-lactams, including last-resort carbapenems [10].

Subclass B1 is the most clinically relevant of the MBLs subclasses, and includes the IMP- (imipenemase) and VIM-types (Verona integrin encoded MBL), which are targets in our study, as well as the NDM-type (New Delhi MBL) [11,12]. The MBL IMP variants have been reported with increasing frequency since their first detection in a *Pseudomonas aeruginosa* isolate in Japan in 1990 [13]. VIM-1 was first identified in Italy a few years later, in 1997, in a *P. aeruginosa* strain. This was followed, soon after, by the isolation of an allelic variant (VIM-2) in France. VIM-1 possesses the broadest range of substrate hydrolysis and can degrade nearly all *β*-lactams, including cephamycins, oxacephamycins, and carbapenems [14], and thus represents the first cause for the failure of carbapenem treatment in bacterial infections [15,16]. MBLs can hydrolyse all the classes of *β*-lactams with the exception of monobactams. In addition, the genes that encode MBLs are carried on plasmids, thus allowing them to spread to other pathogens through horizontal gene transfer [17].

While several inhibitors for SBLs are currently available in therapy, no inhibitors for MBLs have been approved so far, and only a few molecules are now in clinical trials [18,19,20]. It is important to emphasize that, in clinical strains, BLs of several classes, SBLs as well as MBLs, are often coproduced, thus expanding bacterial resistance to all available *β*-lactams and leaving few therapeutic options available [21]. Cross-class BL inhibitors (BLIs) that can synergise *β*-lactams against several BLs would undoubtedly be beneficial in bacterial resistant infections and would help the rehabilitation of many antibiotics that are now ineffective. However, despite its attractiveness, the design of cross-classes BLIs is complicated by the structural and mechanistic differences that characterize SBLs and MBLs. Their binding site architectures are different, challenging the design of broad-spectrum inhibitors. In MBLs’ binding site two zinc ions, Zn1 and Zn2, are generally present and maintained in place by an extensive coordination network. In the apo form of both VIM-1 and IMP-1, a catalytic water (wat1) bridges Zn1 to Zn2, while another conserved water molecule (wat2) coordinates the Zn2 atom (Figures S1 and S2). Conversely, in KPC-2, like in all SBLs, the process involves the catalytic Ser70, which performs a nucleophilic attack on the *β*-lactam core. The *β−*lactam substrate is further stabilized in the active site by an extensive network of interactions [22,23,24]. The first inhibitor active against both SBLs and MBLs classes has, only recently, reached clinical trials [19,20].

Among metallo-BLIs (MBLIs) so far designed, bisthiazolidines, thiols, tetrazoles, sulphonamides, succinic acids, hydroxamates, and boronic acids are recurrent moieties [11,25,26,27,28,29]. In particular, sulfur-containing molecules that are able to interfere, in the binding site, with the hydrolytic water and the Zn-ion network of interactions represent chemical entities that have been widely characterized as MBLIs. Moreover, triazole-thio based compounds have frequently been identified in virtual screening campaigns as micromolar inhibitors of NDM-1 [30,31], IMP-1 [32] and VIM-2 [33].

Interestingly, despite the effectiveness of this scaffold and the pressing need to strike SBLs and MBLs simultaneously, there has been no concerted effort to extend the inhibitory profile of triazole-thiol derivatives against other BL classes, i.e., class A carbapenemases, thus far.

With this in mind, we have selected the 4-amino-1,2,4-triazole-3-thione as the starting scaffold for the design and synthesis of a small library of new possible MBLIs [30,31,32,33,34,35]. The 14 compounds in the library were designed to extend their activity towards class A SBL KPC-2, while maintaining their affinity vs MBLs.

The broad-spectrum profile of the obtained derivatives was validated in vitro against one SBL representative, class A carbapenemases KPC-2, and against two representatives of the MBLs subclass B1, VIM-1 and IMP-1. Moreover, several compounds in the synthesized library have been evaluated for their ability to synergize *β*-lactams in biological tests against clinical strains overexpressing targeted BLs.

## 2. Results and Discussion

### 2.1. Compounds Design and Synthesis

A library of 14 new compounds that share the 4-amino-2,4-dihydro-3H-1,2,4-triazole-3-thione scaffold has been designed and synthesized. Our approach recognizes that, while the thione is fundamental for binding to MBLs through the coordination of the Zn ions (the triazole-thio moiety is common in several MBLs inhibitors [30,31,32,33]), the triazole itself may provide the KPC-2 binding site with the necessary electron-donor requirements for its interactions with key residues such as Ser70, Lys73, Ser130 and Asn132. Moreover, triazole is a bioisostere of the carboxylic group and is, thus, able to make favourable interactions in the deepest part of the class A BL binding site [36,37,38,39].

**Scheme 1:**
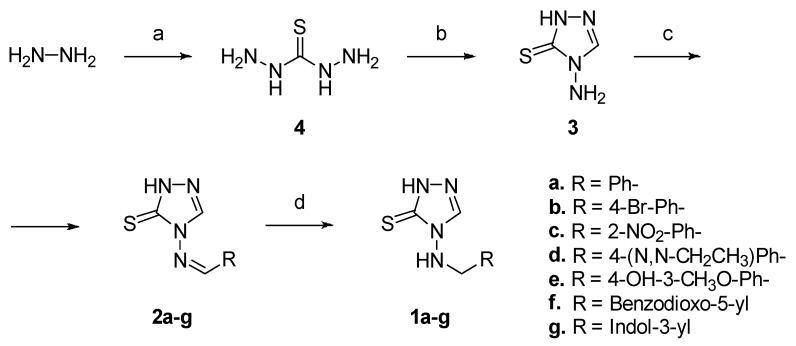
Reagents and conditions: (a) CS_2_, H_2_O, refl., 3h, 50% yield; (b) formic acid (1 mL per gram of **4**), refl., 45 min, 87% yield; (c) aryl carboxaldehyde (1.1 eq.), 35% HCl (cat.), EtOH, refl., 4–12h, yield: 76% (for **2a**), 85% (for **2b**), 67% (for **2c**), 62% (for **2d**), 87% (for **2e**), 79% (for **2f**), 70% (for **2g**); (d) NaBH_4_ (8 eq.), 80% EtOH (*v/v*), 0 °C to r.t. 6–24h, yield: 90% (for **1a**), 87% (for **1b**), 75% (for **1c**), 83% (for **1d**), 75% (for **1e**), 87% (for **1f**), 81% (for **1g**).

The small series of amines (**1a–g**) and the corresponding imine (**2a–g**) derivatives, variously decorated to reach additional active-site residues in SBLs and MBLs, were obtained according to Scheme 1.

The 4-amino-2,4-dihydro-3H-1,2,4-triazole-3-thione (**3**) moiety was synthesized via the reaction of aqueous hydroxylamine with carbon disulfide in refluxing water for 3 h to give hydrazinecarbothiohydrazide (**4**) first. This was followed by cyclization with formic acid under reflux for 45 min. The condensation of **3** with the appropriate aryl carboxaldehyde in refluxing ethanol in presence of hydrochloric acid, used as catalyst, gave the corresponding imines **2a–g** in good yields. Bidimensional NMR analysis (NOESY) confirmed an *E*-configuration at the imine double bond (see Supporting Information). Finally, the reduction of the imine **2a–g** with NaBH_4_ in 80% aqueous ethanol for 6–24 h, led to the respective final amines **1a–g** in good yields, **and** avoided the oxidation of thiol to disulphide, as previously reported (Scheme 1) [40]. Both imines **2a–g** and amines **1a–g** were obtained and isolated in the 1,2,4-triazole-3-thione tautomeric form as confirmed by ^1^H NMR spectroscopy. Our compounds showed the characteristic proton signals for NH groups in the 13–14 ppm range, in accordance with the spectroscopic thiol-thione tautomeric studies reported in the literature [41,42,43,44].

### 2.2. Compounds In-Vitro Validation vs. SBLs and MBLs

The compounds were screened in vitro for their inhibitory activity against carbapenemase MBLs VIM-1 and IMP-1, and SBL KPC-2, using a spectrophotometric assay (Table S1). Compounds **1d**, **1f**, **2b** and **2g** showed a weak, but promising broad-spectrum inhibitory activity against the three targeted BLs (Table 1). The detected inhibitory activities against MBLs and SBLs define the compounds in the library as micromolar but, also, as able to target cross-class BLs, that are notably mechanistically and structurally different. The obtained results clearly indicate that the candidates follow the intended direction of designing broad-spectrum inhibitors.

### 2.3. Molecular Docking and Molecular Dynamics

**Figure 1:**
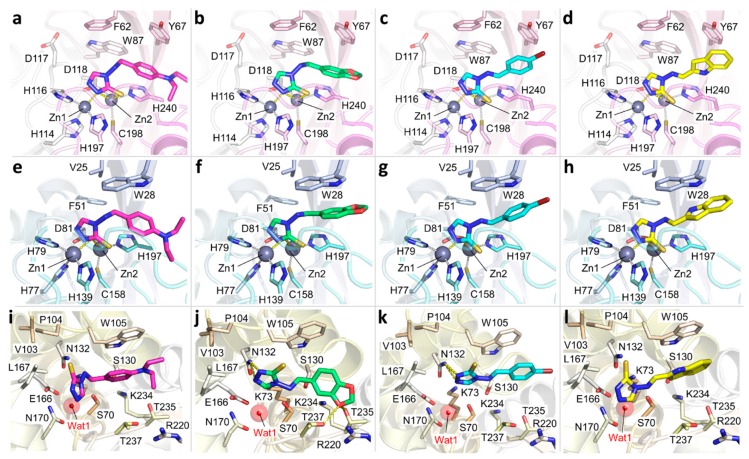
Docking pose of the compounds in VIM-1, IMP-1 and KPC-2. (**a**–**d**) Compounds **1d**, **1f**, **2b** and **2g** in VIM-1 binding site, respectively. (**e**–**h**) Compounds **1d**, **1f**, **2b** and **2g** in IMP-1 binding site, respectively. (**i**–**l**) Compounds **1d**, **1f**, **2b** and **2g** in KPC-2 binding site, respectively. The position originally occupied by the catalytic water wat1 in KPC-2 has been indicated. Even when the water was not retained in the docking simulations, none of the compounds occupied its position, nor was it able to generate valuable contact with it. Proteins are displayed as cartoon (VIM-1, magenta; IMP-1, light blue; KPC-2 dark yellow), zinc atoms are solid grey spheres and their coordination bonds are represented as grey dashed lines. The side chains of the relevant residues are shown as sticks and are labelled accordingly; ligands are depicted in thick, bright-coloured sticks. Hydrogen bonds formed by the ligand with the residues lining the pocket are shown as yellow dashed lines. The catalytic water in KPC-2 is labelled and shown as a red sphere. For clarity, residues have been numbered according to the PDB.

The four compounds were docked in the protein active site, providing valuable indications on the orientation of thio-triazole candidates in carbapenemases (Figure 1). Even if the compounds were isolated as thiones, we assumed, as detailed hereafter, that the presence of the zinc ions in MBLs binding site favours the shifting of the tautomeric equilibrium towards the thiol form and the consequent deprotonation, with the generation of a thiolate able to properly coordinate the Zn ions. Differently, in SBLs the thione form was accounted as the most favourite.

#### 2.3.1. MBLs

The binding pose of these scaffolds in MBL binding site is conserved. The zinc-chelating power derives from their anionic nature: the delocalization of the negative charge between the 2-nitrogen and the non-ring sulfur may indicate the prevalence of a thiolate, rather than a thione, as supported by crystallographic evidence [40,41] and by recent works [34,46]. These observations led us to simulate the compounds as thiolates when docked in the MBLs. All four compounds present a common orientation in MBLs, with the triazole nitrogen in position 2 and the thiolate occupying the position of wat1 and wat2, thus coordinating Zn1 and Zn2, respectively (Figure 1a–h). As mentioned previously, this motif had already been observed in the crystallographic structures of L1 di-zinc MBL, in complex with 1,2,4-triazol-3-thiol (PDB code 2hb9 and 5dpx) [31,47]. Indeed, Zn1 is coordinated by one nitrogen, while Zn2 is coordinated by the deprotonated sulfur atom in both structures (Figure S3). Other than the coordination of Zn1 and Zn2, the interactions with the pocket are mainly hydrophobic and involve residues located in the L3 loop or flanking the binding site, namely Phe62, Tyr67, Trp87 and His240 in VIM-1, and Val25, Trp28, Phe51 and His197 in IMP-1. In particular, in VIM-1, compound **1d** establishes π-π interactions with Tyr67 and His240 through the aromatic substituent (Figure 1a). Compound **1f** maintains the same orientation (Figure 1b), as do **2b** and **2g**, which, however, orient the aromatic substituents more towards Tyr67 than His240 (Figure 1c,d, respectively). In this pose, a substantial portion of the ligand points towards the solvent-exposed side of the hinge, allowing quite bulky substituents to be accommodated. Interestingly, the most active compounds **2b** and **2g** both present an imine double bond in the *E* configuration, which possibly provides the aromatic group with the proper orientation to form closer π-π interactions with Tyr67.

In order to better investigate the binding pose and the binding path of the most promising candidate, **2b**, in VIM-1, we performed 15 Molecular Dynamics (MD) docking replicas (20 ns each). Even though the MD docking shed light on the dynamic path the ligand experiences before binding VIM-1, no relevant information was added to the rigid docking results: the width/openness of the binding cavity offered straightforward access to **2b**, and hydrophobic interactions with Tyr67 and His240 were principally responsible for stabilising the ligand in the binding site. The MD docking in VIM-1 therefore almost identically reflected the interactions and observations that had already been reported in rigid docking studies, and hence corroborate the reliability of the static analyses in MBLs.

The most probable pose assumed by the ligands in IMP-1 binding site largely resembled the one in VIM-1. Again, the zinc ions are coordinated by the thiolate and by the triazole nitrogen, and the rest of the ligand forms π-π interactions with Trp28, which replaces Tyr67 in VIM-1 (Figure 1e–h). The best inhibition was obtained for compound **2g**, which forms an almost perfect contact with Trp28.

#### 2.3.2. KPC-2

Given the absence of the zinc ions, the compounds were modelled in the thione form when docked in the KPC-2 binding site. In accordance with the architecture that this binding cavity presents, the docking returned a ligand orientation in which the triazole moiety always sinks into the active site, while the substituents introduced at position 4 points towards the opening of the binding site, which is delimited by Trp105 (Figure 1i–l). Compound **1d**, the least active, only forms a π-π interaction with Trp105. On the other hand, compound **1f** loses this contact with Trp105, but H-bonds to Asn132 through the triazole nitrogens at positions 1 and 2, and to Thr235 and Thr237 by means of a benzodioxole oxygen. Compounds **2b** and **2g** also show similar inhibition activity and a similar binding mode. They both form good π-π interactions with Thr235; with that of **2g,** possibly being stronger because of the larger aromatic system. Compound **2b** also H-bonds Asn132, similarly to **1f**. In general, the similarities of the poses satisfactorily explain the comparable inhibition activity of the four compounds. The H-bonds formed by some of them with the residues lining the pocket open the way for the optimization of these derivatives to provide them with substituents able to interact more extensively within KPC-2 active site. Indeed, while the hydrophobic requirements of the binding site are well met by the compound aromatic regions, the number of polar interactions should be increased to further improve the binding affinity. In particular, compounds could be functionalized to contact Arg220 via a stronger electrostatic interaction. Furthermore, polar substituents could be attached to the aromatic portion to better reach the residues lining the oxyanion hole, that is Thr235 and Thr237.

### 2.4. Determination of Minimum Inhibitory Concentration (MIC) against Clinical Strains

To investigate the ability of the compounds to reach the periplasmic space, where BLs are secreted and concentrated in Gram-negative bacteria, and to synergically protect β-lactam antibiotics from BLs hydrolysis, the minimum inhibitory concentration (MIC) values were determined against clinical strains overexpressing BLs targets of our studies (Table S2). Unfortunately, the obtained MIC showed no synergistic effect for none of the tested compounds, except for **1a, 1c, 2c** and **2g** which very slightly potentiated meropenem (MEM) activity.

It is important to consider that, in clinical strains, multiple mechanisms of resistance are co-present and employed by bacteria, thus complicating the interpretation of the obtained results. At the same time the necessity to validate designed compounds against their clinical bacterial targets is of critical importance for the further effective selection of candidates for hit-to-lead optimization.

## 3. Conclusions

A library of 14 derivatives has been designed, synthesized and tested, both, in vitro and in biological tests against MBLs and SBLs.

The most active compounds showed weak, but cross-class, inhibitory activity against all targeted BLs, confirming the possibility to introduce broad-spectrum activity on recurrent moieties targeting only one class. Starting from a scaffold that is widely recognised to bind to the MBLs active site, extended affinity against SBLs was achieved, although only at micromolar potency. Molecular modeling and dynamics experiments have been performed to analyse the binding orientations responsible for the inhibitory activity of the most active compounds. The obtained results may serve as a guide in the necessary hit-to-lead optimization of these derivatives, as the most opportune substitutions that can lead to more extensive interactions within BLs active sites will be sought out.

So far, the broad-spectrum inhibitors that have been designed to be active against SBLs and MBLs have mimicked the tetrahedral intermediates that are common to all BL classes [18,19,20].

This often implies that a covalent mechanism of inhibition is involved, at least for SBLs [26,48]. In our case, the developed molecules possess a micromolar affinity towards SBLs and MBLs and were non-covalent inhibitors of SBLs. This was possible thanks to detailed 3D structural information, known mechanisms of action and computational methodologies.

The best molecules will now undergo X-ray crystallographic studies to support further hit-to-lead optimisation processes.

## 4. Materials and Methods

### 4.1. Chemistry

All commercially available chemicals and solvents were reagent grade and were used without further purification unless otherwise specified. Reactions were monitored by thin-layer chromatography on silica gel plates (60F-254, E. Merck) and visualized with UV light, cerium ammonium sulphate or alkaline KMnO_4_ aqueous solution. The following solvents and reagents have been abbreviated: ethyl ether (Et_2_O), dimethyl sulfoxide (DMSO), ethyl acetate (EtOAc), dichloromethane (DCM), dimethyl formamide (DMF), methanol (MeOH), and ethanol (EtOH). NMR spectra were recorded on a Bruker 400 spectrometer with ^1^H at 400.134 MHz and ^13^C at 100.62 MHz. Proton chemical shifts were referenced to the solvent residue peak. Chemical shifts are reported in parts per million (ppm, δ units). Coupling constants are reported in Hertz (Hz). Splitting patterns are designed as s, singlet; d, doublet; t, triplet; q quartet; dd, double doublet; m, multiplet; b, broad. Mass analysis was performed on an Agilent 1200 series LC system coupled to an Agilent 6310A Ion Trap mass spectrometer (LC/MS) (Agilent Technologies, Milan, Italy). Melting points were recorded on a Stuart, SMP3 (Barloworld Scientific Limited Stone, Staffordshire, UK) and are uncorrected. Elemental analysis was performed on a C,H,N,S CE Instruments EA 1110. All the compounds showed a level of purity above 95% by NMR, ESI-MS analysis, melting point and elemental analysis. ^1^H and ^13^C NMR Spectra of all compounds are reported in Supporting Information.

#### 4.1.1. General Procedure for the Synthesis of Amines 1a–g

To a stirred solution of imine **2a–g** (1 eq.) in 80% aqueous ethanol, NaBH_4_ (8 eq.) was added at 0 °C. The mixture was stirred at room temperature, in a close vessel, until disappearance of the starting materials as stated by TLC (12–72 h). The solvent was removed under reduced pressure and the crude triturated in acetate buffer (pH 5). The solid obtained was collected by filtration, dried and purified by crystallization or column chromatography.

#### 4.1.2. General Procedure for the Synthesis of Imines 2a–g

To a stirred suspension of **3** (1 eq.) in EtOH, the appropriate aryl carboxaldehyde (1.1 eq.) and a drop of 37% aqueous HCl as catalyst were added. The suspension was refluxed until disappearance of the starting materials as stated by TLC (4–12 h). The solution was cooled at room temperature and the solvent partially concentrated to 1–2 mL. By standing overnight at 0 °C, a solid precipitate from the mother solution. The solid was collected by filtration, rinsed with cold EtOH and dried to give the pure desired product.

#### 4.1.3. Spectroscopic Data

4-(benzylamino)-2,4-dihydro-3H-1,2,4-triazole-3-thione (**1a**)

Crystalized from Et_2_O. Pale yellow solid (90% yield). M.p. [138.8–140.2 °C]. ^1^H NMR (DMSO-*d6*) δ 4.26 (d, *J* = 4.3 Hz, 2H), 6.00 (t, *J* = 4.5 Hz, 1H), 7.23–7.33 (m, 5H), 7.80 (s, 1H), 12.53 (bs, 1H). ^13^C NMR (DMSO-*d6*) δ 55.70, 127.64, 128.03 (×2), 129.00 (×2), 136.76, 142.49, 160.25. ESI-MS m/z [M + H]^+^ Calcd. for C_9_H_11_N_4_S^+^: 207.0. Found: 207.1.

4-((4-bromobenzyl)amino)-2,4-dihydro-3H-1,2,4-triazole-3-thione (**1b**)

Crystalized from Et_2_O. White solid (87% yield). M.p. [131.3–132.7 °C]. ^1^H NMR (400 MHz, DMSO-*d6*) δ 4.28 (s, 2H), 6.53 (bs, 1H), 7.29 (d, 2H, *J* = 8.0 Hz), 7.53 (d, 2H, *J* = 8.0 Hz), 8.17 (s, 1H), 13.70 (s 1H). ^13^C NMR (DMSO-*d6*) δ 52.34, 121.21, 131.70 (×2), 131.79 (×2), 136.54, 142.92, 165.63. ESI-MS m/z [M + H]^+^ Calcd. for C_9_H_10_BrN_4_S^+^: 285.0. Found: 285.1.

4-((2-nitrobenzyl)amino)-2,4-dihydro-3H-1,2,4-triazole-3-thione (**1c**)

Purified by column chromatography using DCM:Et_2_O 8:2 as mobile phase. Yellow solid (75% yield). M.p. [140.1–141.2 °C]. ^1^H NMR (DMSO-*d6*) δ 4.62 (d, 2H, *J* = 4.57 Hz), 6.95 (t, 1H, *J* = 4.46 Hz), 7.58 (t, 1H, *J* = 8.0 Hz), 7.62 (d, 1H, *J* = 8.0 Hz), 7.69 (dt, 1H, *J* = 7.72, 1.0 Hz), 8.01 (dd, 1H, *J* = 8.0 Hz, 1.0 Hz), 8.18 (s, 1H), 13.70 (bs, 1H). ^13^C NMR (DMSO-*d6*) δ 50.23, 125.17, 129.64, 131.90, 132.04, 133.95, 142.43, 149.47, 165.66. ESI-MS m/z [M + H]^+^ Calcd. for C_9_H_11_N_5_O_2_S^+^: 252.0. Found: 252.1.

4-((4-(diethylamino)benzyl)amino)-2,4-dihydro-3H-1,2,4-triazole-3-thione (**1d**)

Crystalized from Et_2_O. Yellow solid (83% yield). M.p. [140 °C with dec.]. ^1^H NMR (DMSO-*d6*) δ 1.14 (t, 6H, *J* = 8.0 Hz), 3.36–3.39 (m, 4H), 4.62 (d, 2H), 6.67 (d, 2H, *J* = 8.0 Hz), 6.94–6.97 (m, 3H), 7.18 (s, 1H), 13.69 (bs, 1H). ^13^C NMR (DMSO-*d6*) δ 12.29 (×2), 45.30 (×2), 55.81, 115.65 (×2), 121.86 (×2), 138.13, 142.47, 148.34, 160.22. ESI-MS m/z [M + H]^+^ Calcd. for C_13_H_20_N_5_S^+^: 278.1. Found: 278.2. Anal. Calcd for C_13_H_19_N_5_S: C, 56.29; H, 6.90; N, 25.25; S, 11.56. Found: C, 56.32; H, 6.87; N, 25,20; S, 11.70.

4-((4-hydroxy-3-methoxybenzyl)amino)-2,4-dihydro-3H-1,2,4-triazole-3-thione (**1e**)

Purified by column chromatography using DCM:Et_2_O 8:2 as mobile phase. White solid (75% yield). M.p. [131.1–132.3 °C]. ^1^H NMR (DMSO-*d6*) δ 3.75 (s, 3H), 4.16 (s, 2H), 6.55 (bs, 1H), 6.63 (dd, 1H, *J* = 1.9, 8.0 Hz), 6.69 (d, 1H, *J* = 8.0 Hz), 6.88 (d, 1H, *J* = 1.9 Hz), 8.08 (d, 1H, *J* = 1.9 Hz), 8.94 (s, 1H), 13.67 (s, 1H). ^13^C NMR (DMSO-*d6*) δ 53.07, 55.94, 113.33, 115.64, 122.00, 127.54, 142.80, 146.53, 147.98, 165.44. ESI-MS m/z [M + H]^+^ Calcd. for C_10_H_13_N_5_O_2_S^+^: 253.1. Found: 253.2.

4-((benzo[d][1,3]dioxol-5-ylmethyl)amino)-2,4-dihydro-3H-1,2,4-triazole-3-thione (**1f**)

Crystalized from Et_2_O. White solid (87% yield). M.p. [160.2–161.7 °C]. ^1^H NMR (DMSO-*d6*) δ 4.18 (d, 2H, *J* = 4.2 Hz), 6.00 (s, 1H), 6.63 (t, 1H, *J* = 4.2 Hz), 6.70 (d, 1H, *J* = 7.8 Hz), 6.84 (d, 1H, *J* = 7.9 Hz), 6.92 (s, 1H), 8.14 (s, 1H), 13.67 (bs, 1H). ^13^C NMR (DMSO-*d6*) δ 52.91, 101.40, 108.50, 109.67, 122.83, 130.71, 142.85, 147.17, 147.79, 165.50. ESI-MS m/z [M + H]^+^ Calcd. for C_10_H_11_N_4_O_2_S^+^: 251.0. Found: 251.1. Anal. Calcd for C_10_H_10_N_4_O_2_S: C, 47.99; H, 4.03; N, 22.39; S, 12.81. Found: C, 47.85; H, 4.10; N, 22.45; S, 12.77.

4-(((1H-indol-3-yl)methyl)amino)-2,4-dihydro-3H-1,2,4-triazole-3-thione (**1g**)

Crystalized from MeOH:Et_2_O. Brown solid (81% yield). M.p. [171.5–173.2 °C]. ^1^H NMR (DMSO-*d6*) δ 4.53 (d, 2H, *J* = 4.2 Hz), 6.62 (t, 1H, *J* = 4.2 Hz), 7.01–7.05 (m, 3H), 7.40 (d, 1H, *J* = 8.0 Hz), 7.54 (d, 1H, *J* = 8.0 Hz), 8.14 (s, 1H), 8.25 (s, 1H), 13.60 (bs, 1H). ^13^C NMR (DMSO-*d6*) δ 49.79, 110.86, 112.91, 119.5, 119.80, 121.86, 123.59, 127.33, 136.35, 139.37, 167.21. ESI-MS m/z [M + H]^+^ Calcd. for C_11_H_12_N_5_S^+^: 246.1. Found: 246.2.

4-(benzylideneamino)-2,4-dihydro-3H-1,2,4-triazole-3-thione (**2a**)

White solid (76% yield). M.p. [175.3–176.7 °C], lit. [175–176 °C] [49]. ^1^H NMR (DMSO-*d6*) δ: 7.56–7.63 (m, 3H), 7.86–7.90 (m, 2H), 8.95 (s, 1H), 9.47 (s, 1H), 13.95 (bs, 1H). ^13^C NMR (DMSO-*d6*) δ: 129.02 (×2), 129.65 (×2), 132.98, 136.62, 138.65, 156.84, 161.54. ESI-MS m/z [M + H]^+^ Calcd. for C_9_H_9_N_4_S^+^: 205.1. Found: 205.2.

4-((4-bromobenzylidene)amino)-2,4-dihydro-3H-1,2,4-triazole-3-thione (**2b**)

Pale yellow solid (85% yield). ^1^H NMR (CDCl_3_) δ: 7.78–7.83 (m, 4H), 8.95 (s, 1H), 9.46 (s, 1H), 13.96 (s,1H). ^13^C NMR (CDCl_3_) δ: 128.78, 130.77 (×2), 132.30, 132.78 (×2), 138.43, 159.97, 163.55. ESI-MS m/z [M + H]^+^ Calcd. for C_9_H_8_BrN_4_S^+^: 283.0. Found: 283.1. Anal. Calcd for C_9_H_7_BrN_4_S: C, 38.18; H, 2.49; N, 19.79; S, 11.32. Found: C, 38.12; H, 2.55; N, 19.80; S, 11.30.

4-((2-nitrobenzylidene)amino)-2,4-dihydro-3H-1,2,4-triazole-3-thione (**2c**)

White solid (67% yield). M.p. [172.6–174.1 °C], lit. [175 °C] [50]. ^1^H NMR (DMSO-*d6*) δ: 7.85 (t, 1H, *J* = 8.0 Hz), 7.94 (t, 1H, *J* = 8.0 Hz), 8.12 (d, 1H, *J* = 8.0 Hz), 8.21 (d, 1H, *J* = 8.0 Hz), 8.94 (s, 1H), 10.19 (s, 1H), 14.05 (bs, 1H). ^13^C NMR (DMSO-*d6*) δ 125.46, 127.42, 129.64, 133.31, 134.75, 139.22, 149.15, 155.78, 163.44. ESI-MS m/z [M + H]^+^ Calcd. for C_9_H_9_N_5_O_2_S^+^: 250.0. Found: 250.1.

4-((4-(diethylamino)benzylidene)amino)-2,4-dihydro-3H-1,2,4-triazole-3-thione (**2d**)

Bright yellow solid (62% yield). M.p. [151.7–153.1 °C]. ^1^H NMR (DMSO-*d6*) δ 1.14 (t, *J* = 8.0 Hz, 6H), 3.43 (q, *J* = 8.0 Hz, 4H), 6.78 (d, 2H, *J* = 8.0 Hz), 7.66 (d, 2H, *J* = 8.0 Hz), 8.80 (s, 1H), 9.05 (s, 1H), 13.80 (bs, 1H). ^13^C NMR (DMSO-*d6*) δ 12.85 (×2), 44.34 (×2), 111.51 (×2), 118.39, 131.19, 139.01 (×2), 151.09, 162.78, 163.06. ESI-MS m/z [M + H]^+^ Calcd. for C_13_H_18_N_5_S^+^: 276.1. Found: 276.2.

4-((4-hydroxy-3-methoxybenzylidene)amino)-2,4-dihydro-3H-1,2,4-triazole-3-thione (**2e**)

White solid (87% yield). H NMR (DMSO-*d6*) δ 3.84 (s, 3H), 6.93 (d, *J* = 7.5 Hz, 1H), 7.30 (dd, 1H, *J* = 8.3, 2.0 Hz), 7.44 (s, 1H), 8.84 (s, 1H), 9.26 (s, 1H), 10.02 (bs, 1H), 13.87 (bs, 1H). ^13^C NMR (DMSO-*d6*) δ 56.09, 110.62, 116.12, 123.74, 124.99, 139.13, 148.61, 151.78, 162.84, 162.95. ESI-MS m/z [M + H]^+^ Calcd. for C_10_H_11_N_5_O_2_S^+^: 251.1. Found: 251.2.

4-((benzo[d][1,3]dioxol-5-ylmethylene)amino)-2,4-dihydro-3H-1,2,4-triazole-3-thione (**2f**)

White solid (79% yield). ^1^H NMR (DMSO-*d6*) δ 6.11 (s, 2H), 7.01 (d, *J* = 7.9 Hz, 1H), 7.20 (dd, 1H, *J* = 8.12, 1.54 Hz), 7.34 (d, 1H, *J* = 1.54 Hz), 8.21 (s, 1H), 10.41 (s, 1H). ^13^C NMR (DMSO-*d6*) δ 102.09, 105.57, 108.96, 124.77, 128.93, 140.26, 148.50, 150.11, 154.50, 161.55. ESI-MS m/z [M + H]^+^ Calcd. for C_10_H_9_N_4_O_2_S^+^: 249.0. Found: 249.1.

4-(((1H-indol-3-yl)methylene)amino)-2,4-dihydro-3H-1,2,4-triazole-3-thione (**2g**)

White solid (70% yield). M.p. [251.3–252.8 °C], lit [250–253 °C] [51]. ^1^H NMR (DMSO-*d6*) δ: 7.23 (t, 1H, *J* = 6.65 Hz), 7.28 (t, 1H, *J* = 7.39 Hz), 7.52 (d, 1H, *J* = 8.0 Hz), 8.09 (s, 1H), 8.25 (d, 1H, *J* = 8.0 Hz), 8.86 (s, 1H), 9.38 (s, 1H), 12.01 (bs, 1H) 13.80 (bs, 1H); ^13^C NMR (DMSO-*d6*) δ 110.46, 112.78, 121.90, 122.59, 123.75, 124.64, 135.32, 137.76, 139.06, 159.23, 162.71. ESI-MS m/z [M + H]^+^ Calcd. for C_11_H_10_N_5_S^+^: 244.1. Found: 244.2. Anal. Calcd for C_11_H_9_N_5_S: C, 54.31; H, 3.73; N, 28.79; S, 13.18. Found: C, 54.25; H, 3.70; N, 28.83; S, 13.25.

4-amino-2,4-dihydro-3H-1,2,4-triazole-3-thione (**3**)

A stirred suspension of **4** (1 g, 9.4 mmol) in formic acid (1 mL *per* gram of **4**) is refluxed for 45 min. Thereafter, the suspension is filtered still hot and the filtrate is slowly cooled at room temperature overnight. A grey solid separate from the solution. The solid is collected by filtration and re-crystalized from ethanol to give 926 mg of pink crystals (85% yield). M.p. [165.2–166.7 °C], lit. [167–168 °C] [52]. ^1^H NMR (400 MHz, DMSO-*d6*) δ 5.70 (s, 2H), 8.45 (s, 1H), 13.65 (s, 1H). ESI-MS m/z [M + H]+ Calcd. for C_2_H_5_N_4_S^+^: 117.0. Found: 117.1.

Hydrazinecarbothiohydrazide (**4**)

To a stirred solution of hydrazine hydrate (20 mL) in water (60 mL), carbon disulphide (6 mL) was added dropwise. The mixture was refluxed for 3 h and then slowly chilled down at room temperature overnight. A yellow solid separate from the solution which was collected by filtration and re-crystalized from water to give 2.5 g of yellow-greenish needle crystals. The physical aspect and the melting point of the crystal is in accordance with the literature. [53] ESI-MS m/z [M + H]^+^ calcd. for C_7_H_7_N_4_S^+^: 107.2. Found: 107.3.

### 4.2. Static Docking

Rigid docking studies were carried out with GOLD software version 5.5 (www.ccdc.cam.ac.uk), using default parameters for the genetic algorithm. The CHEMPLP scoring function was then selected to rank the best conformations of ligands in BLs. For each compound, a maximum of 30 poses was generated in VIM-1, IMP-1 and KPC-2 binding sites, respectively. Protomers and tautomers were calculated with MoKa [54] and chosen according to their relative abundance at pH 7.4: in MBLs, the most abundant tautomeric form was the deprotonated, thiolate form, in agreement with reported evidences [31,46,47,55,56,57,58]; in KPC-2 binding site, 1,2,4-triazole-3-thione was the preferred tautomeric state. The binding site in KPC was centred on the hydroxyl atom of residue S70, with a radius of 7 Å. For MBLs, the docking site comprised all residues distant no more than 12 Å from the Zn2 atom.

VIM-1. The available structures of VIM-1 co-crystallized with hydrolyzed meropenem was selected as the target structure (PDB code 5n5i) [59]. The docking simulations were guided by the recently solved crystallographic structure of one thio-triazole derivative complexed with VIM-2 (PDB code 6tgi). The similarity of the protein binding site justifies the approach (Figure S1).

Accordingly, the coordination length was set in a 1.5 Å–1.9 Å range for triazole nitrogen and Zn1 and in a 1.5 Å–2.2 Å range for sulfur and Zn2. Solvent and hydrolyzed substrate were omitted.

IMP-1. X-ray resolved structure of IMP-1 bound to an inhibitor was retrieved from the Protein Data Bank (PDB code 1jjt) [60]. Waters, ligand and maximum separation settings were treated as reported before for VIM-1.

KPC-2. X-ray resolved structure of apo-KPC-2 was retrieved from the Protein Data Bank (PDB code 5ul8), all waters were omitted in docking calculations [23].

### 4.3. MD Docking

The most promising candidate **2b** was submitted to a MD docking in VIM-1 (PDB code 5n5i), to investigate which is the mechanism of binding and which are the residues involved in it. MD docking was set up and performed following the original protocol developed by BiKi Technologies [61]: two subsets (A and B) were defined, with A being the ligand and B the zinc atoms. 15 replicas of 20 ns each were set up, and for each run velocities were reassigned according to a Maxwell-Boltzmann distribution at 300 K. An electrostatic bias was applied until subset A reached a distance of 4 Å from subset B, then the bias was switched-off and the production was carried out as a plain MD until the end of the simulation time.

### 4.4. Proteins Production and Purification

#### 4.4.1. KPC-2

Expression and purification of recombinant KPC-2 have been performed as already reported [62].

#### 4.4.2. VIM-1

The enzyme was purified from *E. coli* BL21 (DE3) transformed with the overexpression vector pet24a(+) carrying blaVIM-1 gene (pBPAQND2009). The bacteria were grown at 37 °C in 1 L of Terrific Broth (Biolife) supplemented with 50 µg/mL of kanamycin (Sigma-Aldrich) until the O.D.600 reached 0.6. The cells were further grown at 27 °C overnight. Afterwards the bacterial suspension was harvested by centrifugation and washed 3 times with 30 mM sodium cacodylate buffer (pH 6.3) (Sigma Aldrich). The dried pellet was subjected to 6 cycles of freeze (−80 °C) / thaw (37 °C) and then resuspended in 115 mL (7 mL/g of cell pellet) of the same buffer supplemented with 100 µM ZnCI2. The cells were disrupted by sonication 10 times for 60 s at 80% power and 50% cycle (UP50H sonicator) and centrifuged 105,000 × g for 60 min at 4 °C. The clarified supernatant was loaded on a High Q column (2.5 × 20 cm; GE Healthcare) equilibrated in 30 mM sodium cacodylate buffer (pH 6.3) supplemented with 100 µM ZnCI2 (CBZ). The bound protein was eluted using a linear NaCI gradient, (0 to 1 M) in CBZ at a flow rate of 3 mL/min. The fractions containing β-lactamase activity were dialyzed against 30 mM HEPES (pH 7.5) supplemented with 100 µM ZnCI2 and concentrated 15-fold by Millipore Amicon 10000 kD centrifuge tubes. The concentrated fractions were loaded on a Superdex 75 column (16 × 100 cm; GE Healthcare, Chicago, IL, USA) previously equilibrated with 30 mM HEPES (pH 7.5) (Sigma-Aldrich, St. Louis, MO, USA) supplemented with 100 µM ZnCI2. The eluted fractions containing VIM-1 β-lactamase was concentrated to 9 mg/mL by centrifugal filtration and stored at −80 °C. The protein concentration was determined by Bradford assay and purity by sodium dodecyl sulfate-polyacrylamide gel electrophoresis (SDS-PAGE).

#### 4.4.3. IMP-1

The codon-optimized wild type blaIMP-1 gene of *Pseudomonas aeruginosa* was purchased by Eurofins Genomics, and directly cloned into pETite N-His SUMO Kan Vector. Gene design and cloning was carried out according to the kit protocol of Expresso^®^ T7 SUMO Cloning and Expression System (Lucigen, Middleton, WI, USA). The correct sequence of the resulting recombinant construct was confirmed by Sanger DNA sequencing (Eurofins Genomics, Ebersberg Germany). Overexpression of the N-His SUMO IMP-1 protein was performed transforming E. coli Lemo21(DE3) cells (NEB). 20 mL of LB medium (25 mg/L kanamycin, 17 mg/L chloramphenicol) were inoculated with fresh colonies and grown at 37 °C overnight. The overnight culture was used to inoculate 1 L of LB medium (25 mg/L kanamycin, 17 mg/L chloramphenicol) grown at 37 °C with shaking. The cells were grown till 0.5 O.D. was reached, induced with 0.2 mM Isopropyl-β-D-1thiogalactopyranoside (IPTG) and incubated overnight at 25 °C. Cell pellet was resuspended in lysis buffer (20 mM Tris-HCl pH 7.5, 150 mM NaCl, 1 mM ZnCl2, 10% glycerol and 20 mM imidazole) supplemented with protease inhibitors cocktail (Roche, Basel, Switzerland), and lysed by French press. The supernatant fractions were isolated from cell debris by centrifugation and N-His SUMO IMP-1 was purified through IMAC (HisTrap HP 1 mL column, GE Healthcare). After an extensive washing with buffer A (20 mM Tris-HCl pH 7.5, 150 mM NaCl, 10% glycerol and 20 mM imidazole), the His-tagged protein was eluted with an increasing gradient of buffer B (20 mM Tris-HCl pH 7.5, 150 mM NaCl, 10% glycerol and 0.5 M imidazole). Fractions containing N-His SUMO IMP-1 were evaluated by SDS-PAGE, and buffer exchanged with PD10 desalting columns (GE Healthcare) in buffer C (20 mM Tris-HCl pH 7.5, 150 mM NaCl and 10% glycerol), to remove imidazole before digestion with SUMO protease. N-His SUMO removal was performed through cleavage of the purified protein, added with 2 mM DTT, with SUMO express Protease (Lucigen), for 2 h at 30 °C. Cleaved IMP-1 was isolated from N-His SUMO, SUMO express Protease (His-tagged) and uncleaved N-His SUMO IMP-1 by IMAC. Most pure fractions of IMP-1 recovered from the flow through, as revealed by SDS-PAGE, were pooled together and concentrated by centrifugal filters (Vivaspin^®^ Turbo 4 10000 MWCO, Sartorius, Göttingen, Germany). A final step of purification was performed by size exclusion chromatography (Superose12 10/300 GL, GE Healthcare) in buffer 30 mM Hepes, 150 mM NaCl pH 7.1.

### 4.5. In Vitro Enzyme Inhibition Assays Against KPC-2, VIM-1 and IMP-1

The half-maximal inhibitory concentration (IC_50_) of synthesized derivatives was determined as follows. Reactions were monitored using a Jasco V-730 spectrophotometer at 485 nm wavelength. Compounds were dissolved in dimethyl sulfoxide (DMSO) to a concentration of 20 mM and stored at −20 °C. Each compound was tested at 5 different concentrations for inhibitory activity vs full-length KPC-2 enzyme, VIM-1 and IMP-1. For class A carbapenemase KPC-2, tests were conducted in 50 mM of PB + 50 mM KCl at pH 7.4 at 25 °C with 0.01% *v/v* Triton X-100, to avoid compound aggregation and promiscuous inhibition [62,63]. For MBLs VIM-1 and IMP-1 tests were conducted in 20mM of HEPES + 100 mM NaCl and ZnSO4 10 μM at pH 7.4 at 25 °C with 0.01% *v/v* Triton X-100. For all targeted proteins, reported substrate nitrocefin was used at 50 μM, (K_m_ 36 μM) for KPC-2, at 25 μM (K_m_ 22 μM) for VIM-1 and at 25 (K_m_ 25 μM) for IMP-1. All the experiments were performed in triplicate. The reaction was typically initiated by adding the enzyme to the reaction buffer last. The IC_50_ values were determined by measuring the rate of hydrolysis of a reporter substrate in the presence of five different inhibitor concentrations at λ 482 nm. The binding affinity *K*_i_ was estimated from the determined IC_50_ by Cheng–Prusoff equation as per competitive inhibition (Table 1 and Table S1) [45]. For targeted enzyme a known, in house broad spectrum inhibitor, was used as control [26,28].

### 4.6. MICs Assays

Susceptibility testing was performed in Mueller Hinton broth and interpreted following the guidelines of the National Committee for Clinical Laboratory Standards [64]. To test the inhibitory activity, the compounds were dissolved in DMSO and further dilutions were done using growth medium. In all cases tests were performed at a final DMSO concentration below 5%, avoiding unwanted DMSO inhibition effect on strains growth (blank test control with DMSO were performed). The MICs of MEM alone and in association were determined against several relevant Gram-negative clinical isolates, selected from the pathogen collection of the Spanish Network for Research in Infectious Pathology (REIPI; Sevilla, Spain). The selection was based on strains capability of producing β-Lactamases of interest for the present study. The MEM: compounds ratio was 1:1 (molar).

## Figures and Tables

**Table 1:** Binding affinity of the most promising broad-spectrum inhibitors against VIM-1, IMP-1 and KPC-2.

Code	Structure	VIM-1 *K*_i_ (µM) ^a^	IMP-1 *K*_i_ (µM) ^a^	KPC-2 *K*_i_ (µM) ^a^
1d	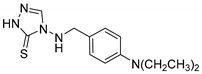	224 ± 11	173 ± 6	263 ± 13
1f	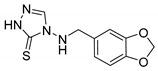	120 ± 4	125 ± 5	138 ± 3
2b	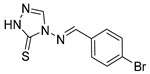	58 ± 2	285 ± 13	158 ± 7
2g	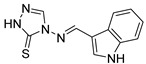	95 ± 3	104 ± 4	139 ± 4

^a^ Estimated *K*_i_ as per competitive inhibitor [45]. All the experiments were performed in triplicate.

## Supplementary Materials

The following are available online at https://www.mdpi.com/1424-8247/13/3/52/s1, Figure S1: Catalytic site of VIM-1; Figure S2: Comparison of VIM-1 and IMP-1 binding site; Figure S3: Crystallographic orientation of a 1,2,4-triazole-3-thiol compound in L1 MBL; Table S1: Enzymatic inhibitor activity of compounds 1a-g and 2a-g against VIM-1, IMP-1 and KPC-2; Table S2: MICs of meropenem in combination with compounds 1a-g and 2a-g; 1H and 13C NMR spectra of compounds 1a-g and 2a-g. (PDF).

## Abbreviations

BLs: β-lactamases; BLI: β-lactamase inhibitor; EtOH: ethanol; IMP: imipenemase; KPC-2: Klebsiella pneumoniae carbapenemase 2; MBL: metallo-β-lactamases, MBLI: metallo-β-lactamase inhibitor; MD: molecular dynamic, MDR: Multi drug resistant, MIC: minimum inhibitory concentration, MEM: meropenem, NOESY: Nuclear Overhauser Effect Spectroscopy, SBLs: serine β-lactamases, TLC: thin layer chromatography, VIM: Verona Integrin-encoded MBL.
