# Supplementary material for: 4-Amino-1,2,4-triazole-3-thione as a Promising Scaffold for the Inhibition of Serine and Metallo-β-Lactamases
Source: Pharmaceuticals (Basel). 2020 Mar 24;13(3):52. doi: 10.3390/ph13030052 (PMC7151704; doi:10.3390/ph13030052)

Article

4-amino-1,2,4-triazole-3-thione as a promising scaffold for the inhibition of serine and metallo *β*-lactamases

Pasquale Linciano [1], Eleonora Gianquinto [2], Martina Montanari [1], Lorenzo Maso [3], Pierangelo Bellio [4], Esmeralda Cebrián-Sastre [5], Giuseppe Celenza [4], Jesús Blázquez [5], Laura Cendron [3], Francesca Spyrakis[2,*] & Donatella Tondi [1,*].

1Department of Life Sciences, University of Modena and Reggio Emilia, Via G. Campi 103, 41125, Modena, Italy;

2Department of Drug Science and Technology, University of Turin, Via P. Giuria 9, 10125, Turin, Italy;

3Department of Biology, University of Padua, Viale G. Colombo 3, 35121, Padua, Italy;

4Department of Biotechnological and Applied Clinical Sciences, University of L'Aquila, via Vetoio 1, 67100 L'Aquila, Italy;

5National Center of Biotechnology-CSIC, Calle Darwin 3, 28049 Madrid, Spain.

***Correspondence: francesca.spyrakis@unito.it (F.S.); donatella.tondi@unimore.it (D.T.)**

Table of content

Figure S1. Catalytic site of VIM-1……………………………………………………….…………………...S2

Figure S2. Comparison of VIM-1 and IMP-1 binding site…………………….……………………… ….S3

Figure S3. Crystallographic orientation of a 1,2,4-triazole-3-thiol compound in L1 MBL…………….S4

Table S1. Enzymatic inhibitor activity of compounds 1a-g and 2a-g against VIM-1, IMP-1 and KPC-2………………………………………………………………………………………………………S5

Table S2. MICs of meropenem in combination with compounds 1a-g and 2a-g…………………………S6

^{1}H and ^{13}C NMR spectra of compounds 1a-g and 2a-g………………………………….. ………..S7

Figure S1. Catalytic site in VIM-1 apo form (PDB code 5n5g). Protein structure is reported in cartoon representation, zinc atoms are shown as grey spheres, bridging water Wat1 and coordinating water Wat2 have been depicted as red spheres and labelled, significant residues coordinating the zinc atoms or lining the binding pocket are shown as sticks, coordination bonds are reported as dashed lines. Loops L3 (red) and L10 (green) are highlighted.

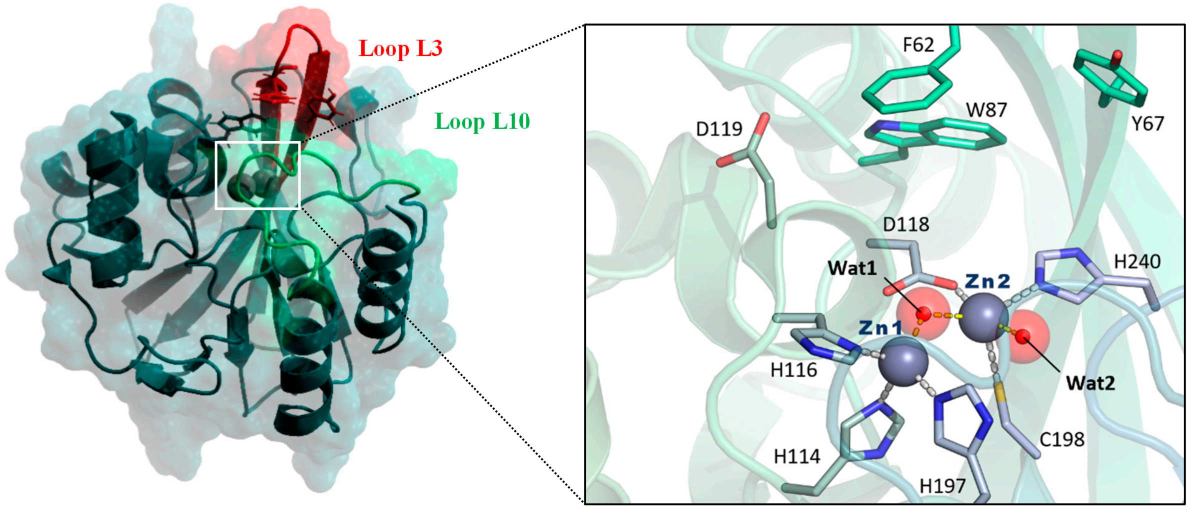

Figure S2. Comparison of VIM-1 (a) and IMP-1 (b) binding site. Protein structure is reported in cartoon representation, zinc atoms are shown as grey spheres, significant residues coordinating the zinc atoms or lining the binding pocket are shown as sticks, coordination bonds are reported as dashed lines.

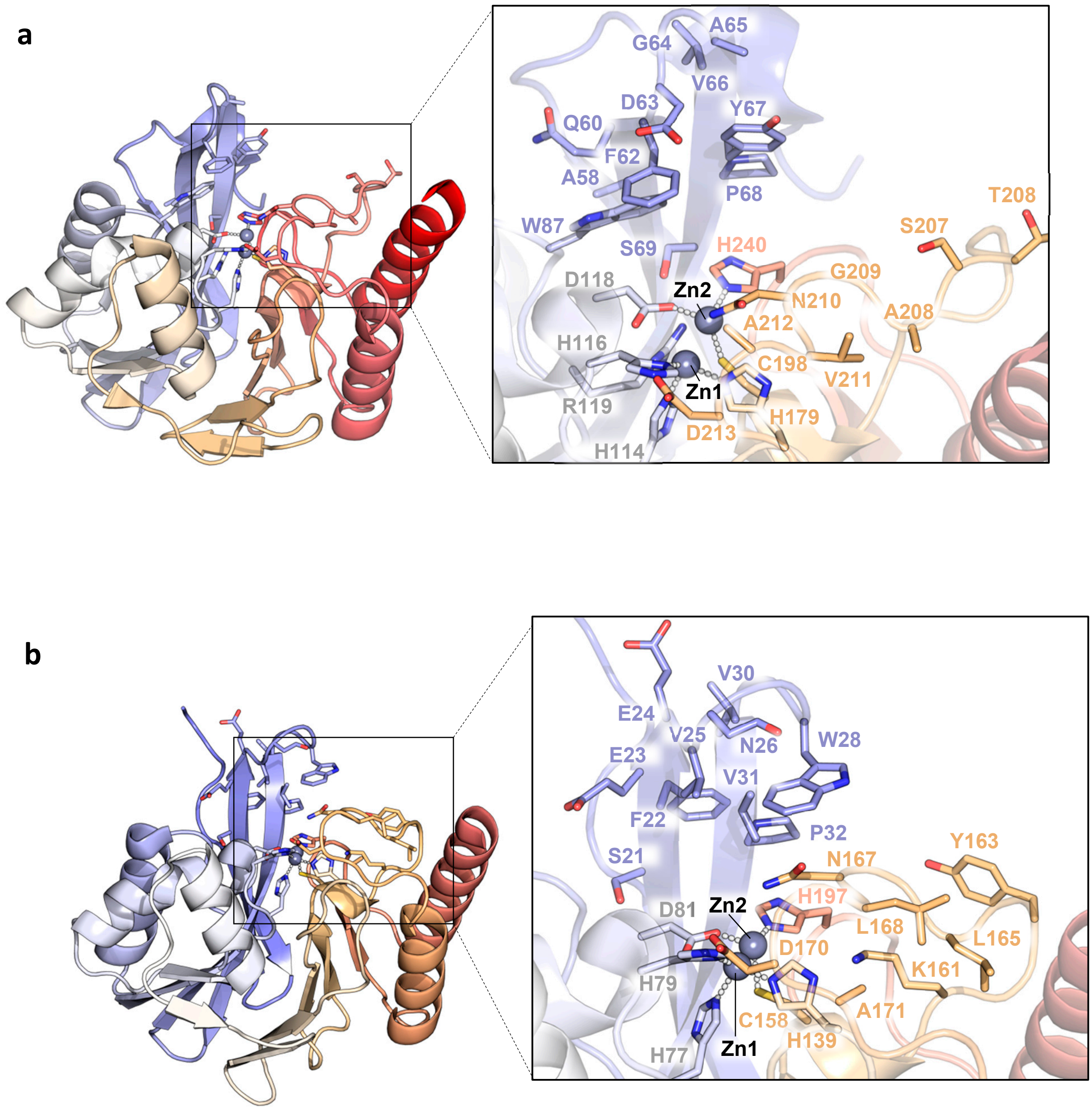

Figure S3. Crystallographic orientation of a 1,2,4-triazole-3-thiol compound in L1 MBL (PDB CODE 5dpx). Protein structure is reported in cartoon representation, zinc atoms are shown as grey spheres, significant residues coordinating the zinc atoms or lining the binding pocket are shown as sticks, coordination bonds are reported as dashed lines.

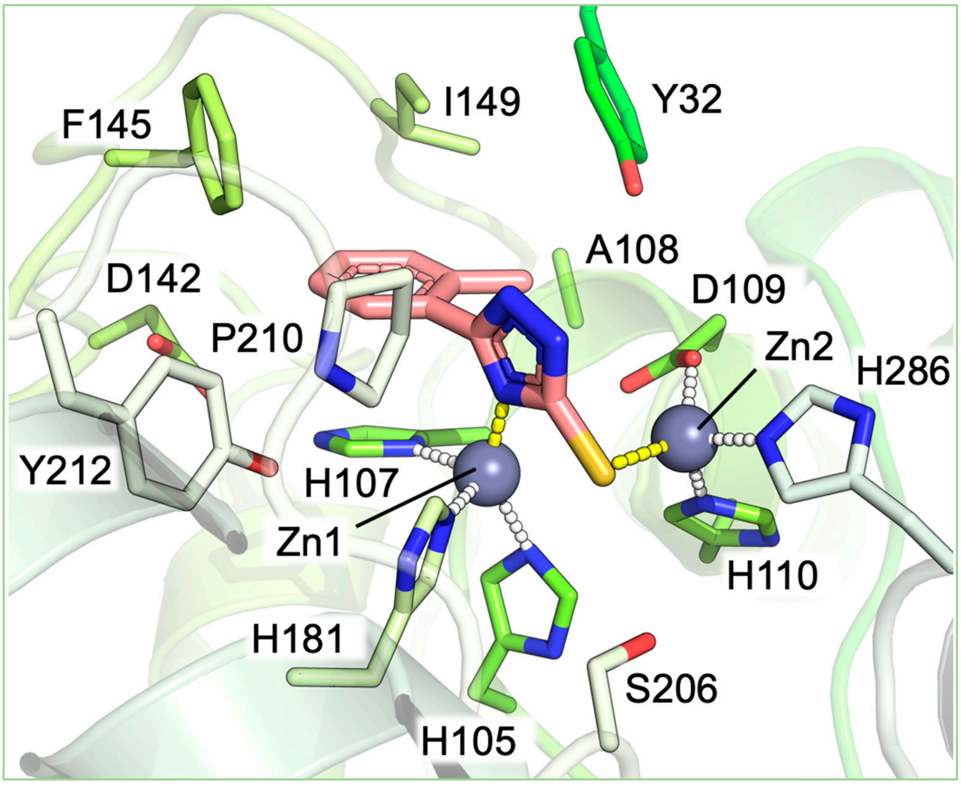

Table S1. Enzymatic Inhibitor Activity of compounds 1a-g and 2a-g against VIM-1, IMP-1 and KPC-2 not included in Table 1.

Code	VIM-1 K_i (µM)	IMP-1 % of inhibition at 200 µM	KPC-2 % of inhibition at 200 µM
**1a**	601	37	20
**1b**	137	51	17
**1c**	182	44	6
**1e**	204	44	15
**1g**	n.t.	n.t	n.t
**2a**	50,3	46	18
**2c**	124	45	4
**2d**	66,9	45	33
**2e**	142	46	7
**2f**	145	40	21

Table S2. MICs of meropenem in combination with compounds 1a-g and 2a-g

Clinical strains of *Klebsiella pneumoniae*, *Pseudomonas aeruginosa* and *P. aeruginosa* producing NDM-1, VIM-2, IMP-28 and KPC-2 were used. The MIC was calculated through the microdilution method using 96-microwell plates. The bacterial inoculum in each well was adjusted at a final dilution of 1:1000 from an overnight culture. Meropenem (MEM) was mixed with every compound maintaining a mole ratio of 1:1 in all the wells.

K. pneumoniae **NDM-1**	**MIC (µg/ml)**
Meropenem (MEM)	512
MEM + 2f	512
MEM + 1a	256 – 512
MEM + 1b	256 – 512
MEM + 1c	512
MEM + 1d	512
MEM + 1e	512
MEM + 1g	512
MEM + 2b	512
MEM + 2c	256
MEM + 2e	256 – 512
MEM + 2g	512

P. aeruginosa **VIM-2**	**MIC (µg/ml)**
Meropenem (MEM)	8
MEM + 2f	8
MEM + 1a	8
MEM + 1b	8
MEM + 1c	8
MEM + 1d	8
MEM + 1e	8
MEM + 1g	8
MEM + 2b	8
MEM + 2c	8
MEM + 2e	8
MEM + 2g	8

P. aeruginosa **IMP-28**	**MIC (µg/ml)**
Meropenem (MEM)	16
MEM + 2f	16
MEM + 1a	16
MEM + 1b	16
MEM + 1c	16
MEM + 1d	16
MEM + 1e	16
MEM + 1g	16
MEM + 2b	16
MEM + 2c	16
MEM + 2e	16
MEM + 2g	16

K. pneumoniae 53A8 **KPC-2**	**MIC (µg/ml)**
Meropenem (MEM)	64
MEM + 1a	32
MEM + 1b	64
MEM + 1c	32
MEM + 1d	64
MEM + 1e	64
MEM + 1g	64
MEM + 2b	64
MEM + 2c	64
MEM + 2e	64
MEM + 2f	64
MEM + 2g	64

K. pneumoniae 53A9 **KPC-2**	**MIC (µg/ml)**
Meropenem (MEM)	64
MEM + 1a	64
MEM + 1b	64
MEM + 1c	64
MEM + 1d	64
MEM + 1e	64
MEM + 1g	64
MEM + 2b	64
MEM + 2c	64
MEM + 2e	64
MEM + 2f	64
MEM + 2g	32

^{1}H and ^{13}C NMR spectra of compounds 1a-g and 2a-g

1a

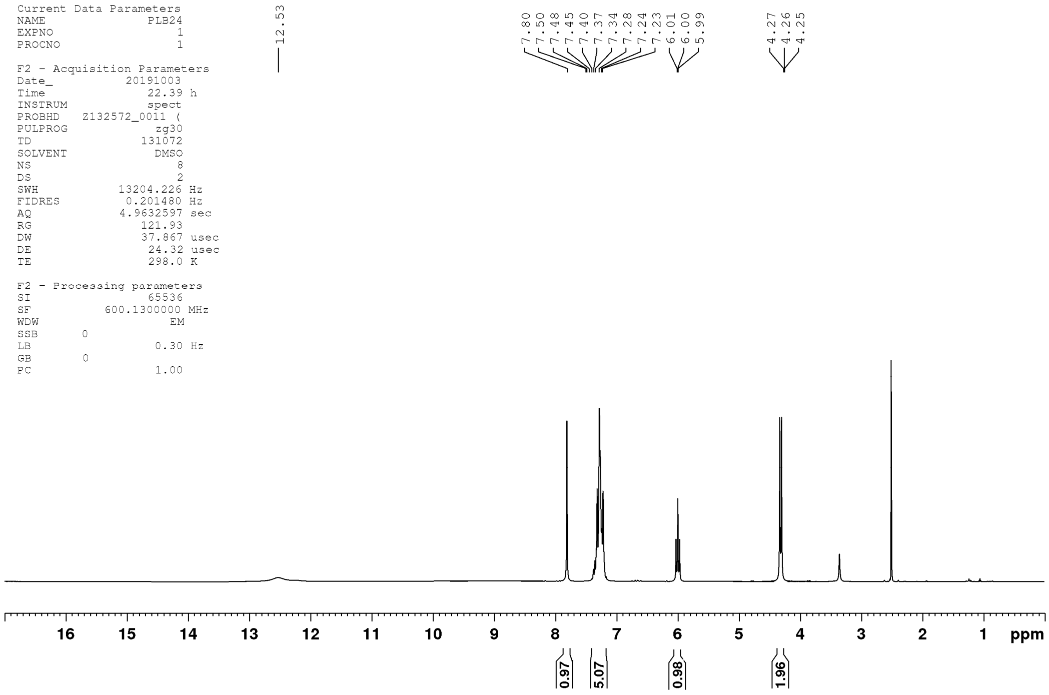

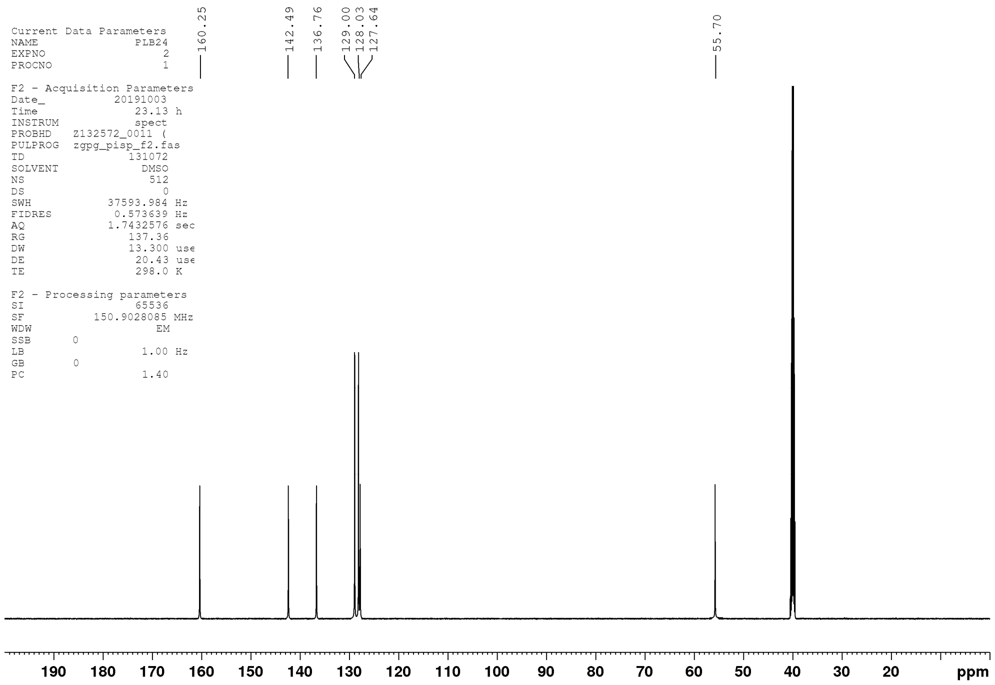

1b

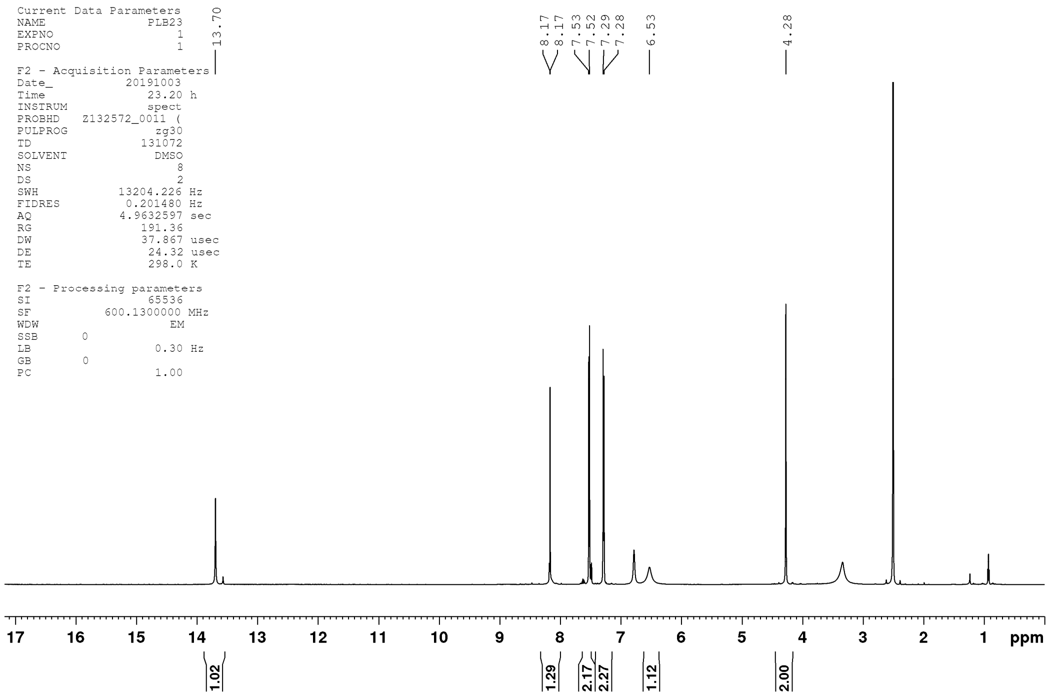
Current Data Parameters
NAME PLB23
EXPNO 1
PROCNO 1
F2 - Acquisition Parameters
Date_ 20191003
Time 23.20 h
INSTRUM spect
PROBHD Z132572_0011 (
PULPROG zg30
TD 131072
SOLVENT DMSO
NS 8
DS 2
SWH 13204.226 Hz
FIDRES 0.201480 Hz
AQ 4.9632597 sec
RG 191.36
DW 37.867 usec
DE 24.32 usec
TE 298.0 K
F2 - Processing parameters
SI 65536
SF 600.1300000 MHz
WDW EM
SSB 0
LB 0.30 Hz
GB 0
PC 1.00
13.70
8.17
8.17
7.53
7.52
7.29
7.28
6.53
4.28
17 16 15 14 13 12 11 10 9 8 7 6 5 4 3 2 1 ppm
1.02
1.29
2.17
2.27
1.12
2.00

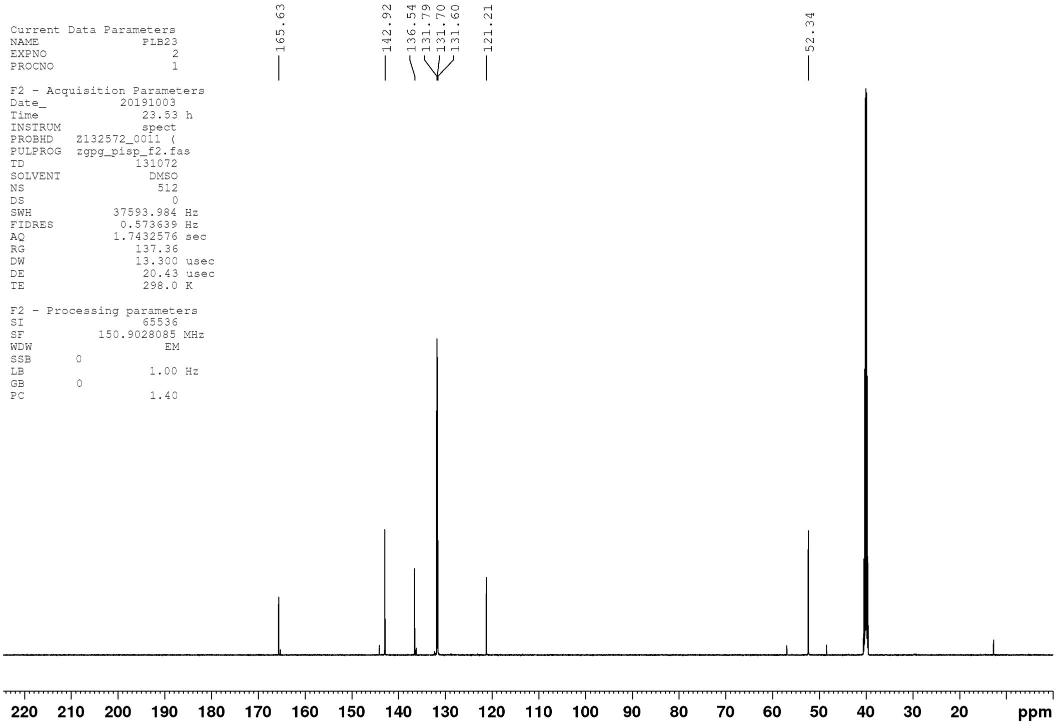
Current Data Parameters
NAME PLB23
EXPNO 2
PROCNO 1
F2 - Acquisition Parameters
Date_ 20191003
Time 23.53 h
INSTRUM spect
PROBHD Z132572_0011 (
PULPROG zgpg_pisp_f2.fas
TD 131072
SOLVENT DMSO
NS 512
DS 0
SWH 37593.984 Hz
FIDRES 0.573639 Hz
AQ 1.7432576 sec
RG 137.36
DW 13.300 usec
DE 20.43 usec
TE 298.0 K
F2 - Processing parameters
SI 65536
SF 150.9028085 MHz
WDW EM
SSB 0
LB 1.00 Hz
GB 0
PC 1.40
165.63
142.92
136.54
131.79
131.70
131.60
121.21
52.34
220 210 200 190 180 170 160 150 140 130 120 110 100 90 80 70 60 50 40 30 20 ppm

1c

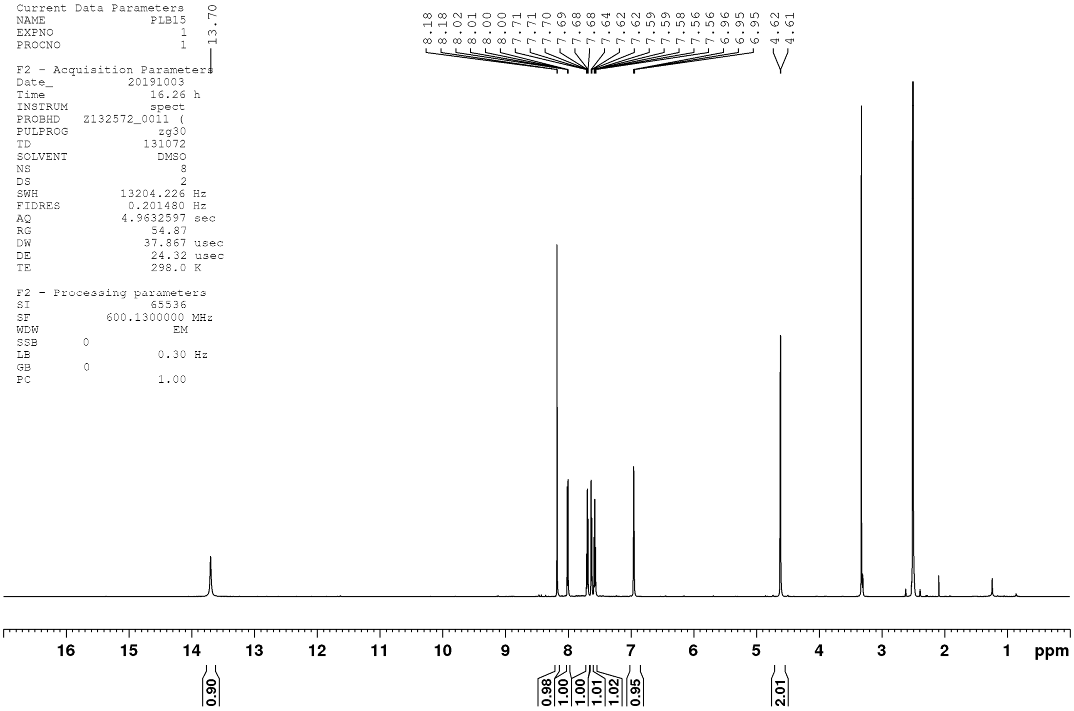
Current Data Parameters
NAME PLB15
EXPNO 1
PROCNO 1
F2 - Acquisition Parameters
Date_ 20191003
Time 16.26 h
INSTRUM spect
PROBHD Z132572_0011 (
PULPROG zg30
TD 131072
SOLVENT DMSO
NS 8
DS 2
SWH 13204.226 Hz
FIDRES 0.201480 Hz
AQ 4.9632597 sec
RG 54.87
DW 37.867 usec
DE 24.32 usec
TE 298.0 K
F2 - Processing parameters
SI 65536
SF 600.1300000 MHz
WDW EM
SSB 0
LB 0.30 Hz
GB 0
PC 1.00
13.70
8.18 8.18 8.02 8.01 8.00 8.00 7.71 7.71 7.70 7.69 7.68 7.68 7.64 7.62 7.62 7.59 7.59 7.58 7.56 7.56 6.96 6.95
4.62 4.61
16 15 14 13 12 11 10 9 8 7 6 5 4 3 2 1 ppm
0.90
0.98 1.00 1.00 1.01 1.02 0.95
2.01

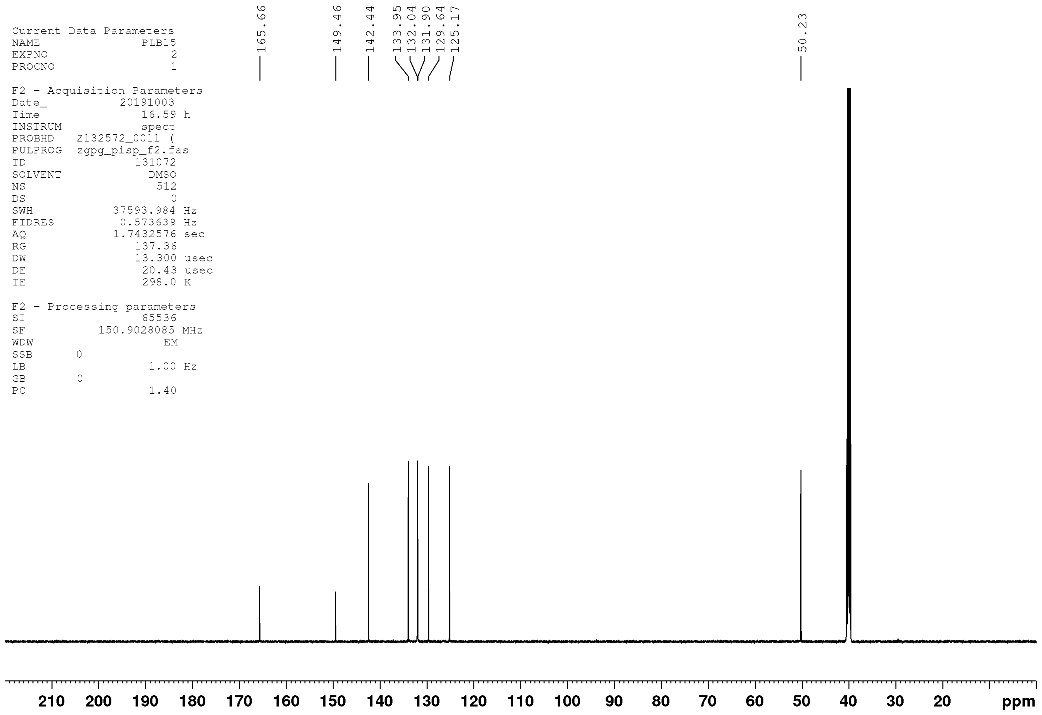
Current Data Parameters
NAME PLB15
EXPNO 2
PROCNO 1
F2 - Acquisition Parameters
Date_ 20191003
Time 16.59 h
INSTRUM spect
PROBHD Z132572_0011 (
PULPROG zgpg_pisp_f2.fas
TD 131072
SOLVENT DMSO
NS 512
DS 0
SWH 37593.984 Hz
FIDRES 0.573639 Hz
AQ 1.7432576 sec
RG 137.36
DW 13.300 usec
DE 20.43 usec
TE 298.0 K
F2 - Processing parameters
SI 65536
SF 150.9028085 MHz
WDW EM
SSB 0
LB 1.00 Hz
GB 0
PC 1.40
165.66
149.46
142.44
133.95 132.04 131.90 129.64 125.17
50.23
210 200 190 180 170 160 150 140 130 120 110 100 90 80 70 60 50 40 30 20 ppm

1d

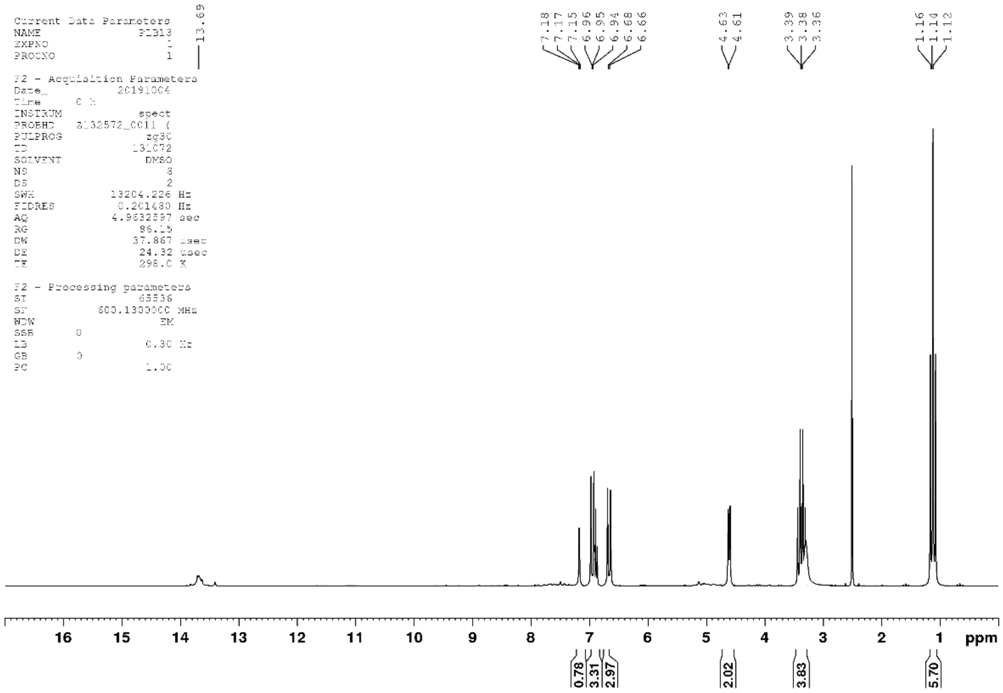
13.69
7.18
7.17
7.15
6.96
6.95
6.94
6.68
6.66
4.63
4.61
3.39
3.38
3.36
1.16
1.14
1.12
16 15 14 13 12 11 10 9 8 7 6 5 4 3 2 1 ppm
0.78
3.31
2.97
2.02
3.83
5.70

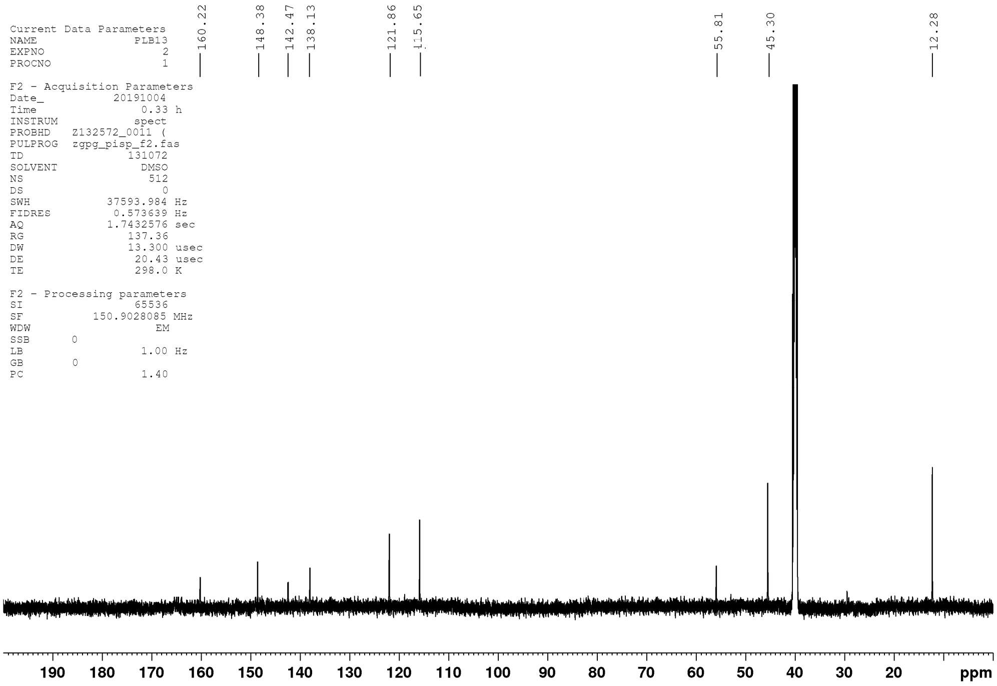
Current Data Parameters
NAME PLB13
EXPNO 2
PROCNO 1
F2 - Acquisition Parameters
Date_ 20191004
Time 0.33 h
INSTRUM spect
PROBHD Z132572_0011 (
PULPROG zgpg_pisp_f2.fas
TD 131072
SOLVENT DMSO
NS 512
DS 0
SWH 37593.984 Hz
FIDRES 0.573639 Hz
AQ 1.7432576 sec
RG 137.36
DW 13.300 usec
DE 20.43 usec
TE 298.0 K
F2 - Processing parameters
SI 65536
SF 150.9028085 MHz
WDW EM
SSB 0
LB 1.00 Hz
GB 0
PC 1.40
160.22
148.38
142.47
138.13
121.86
115.65
55.81
45.30
12.28
190 180 170 160 150 140 130 120 110 100 90 80 70 60 50 40 30 20 ppm

1e

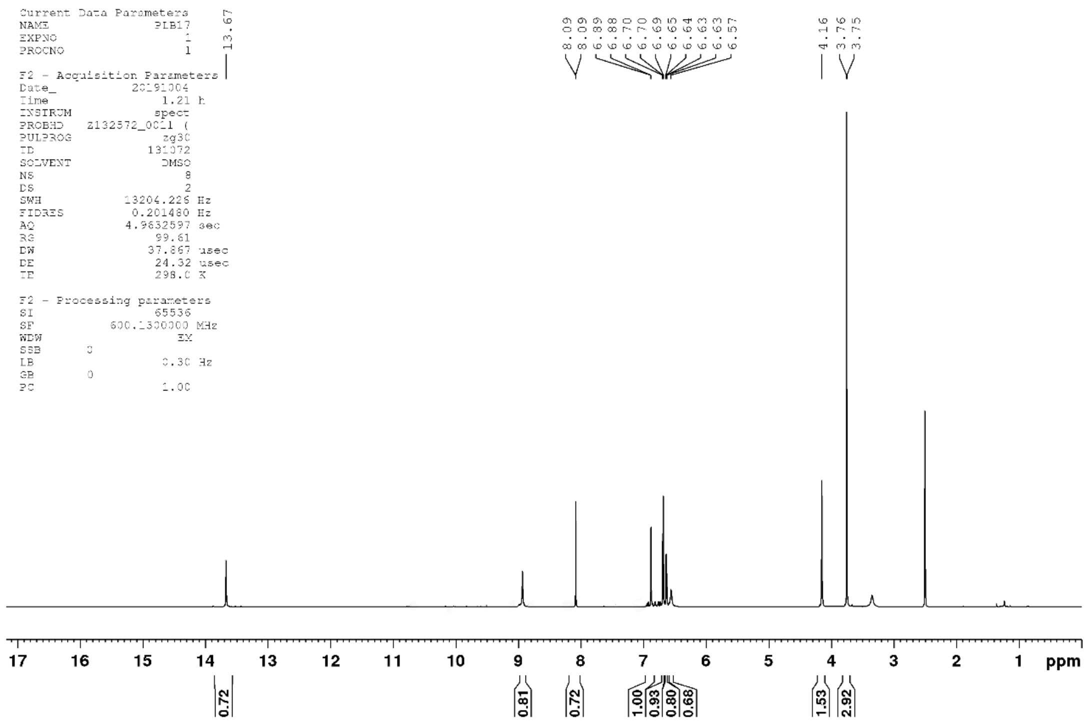
Current Data Parameters
NAME PLB17
EXPNO 1
PROCNO 1
F2 - Acquisition Parameters
Date_ 20191004
Time 1.21 h
INSTRUM spect
PROBHD Z132572_0011 (
PULPROG zg30
TD 131072
SOLVENT DMSO
NS 8
DS 2
SWH 13204.226 Hz
FIDRES 0.201480 Hz
AQ 4.9632597 sec
RG 99.61
DW 37.867 usec
DE 24.32 usec
TE 298.0 K
F2 - Processing parameters
SI 65536
SF 600.1300000 MHz
WDW EM
SSB 0
LB 0.30 Hz
GB 0
PC 1.00
13.67
8.09
8.09
6.89
6.88
6.70
6.69
6.65
6.64
6.63
6.63
6.57
4.16
3.76
3.75
0.72
0.81
0.72
1.00
0.93
0.80
0.68
1.53
2.92
17 16 15 14 13 12 11 10 9 8 7 6 5 4 3 2 1 ppm

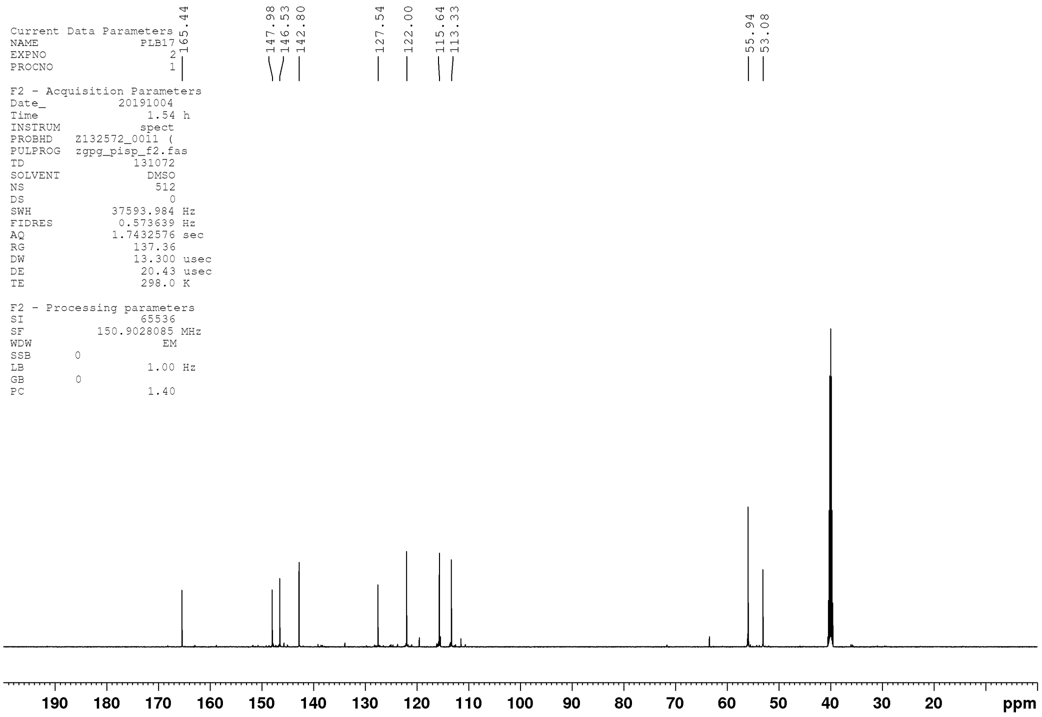
Current Data Parameters
NAME PLB17
EXPNO 2
PROCNO 1
F2 - Acquisition Parameters
Date_ 20191004
Time 1.54 h
INSTRUM spect
PROBHD Z132572_0011 (
PULPROG zgpg_pisp_f2.fas
TD 131072
SOLVENT DMSO
NS 512
DS 0
SWH 37593.984 Hz
FIDRES 0.573639 Hz
AQ 1.7432576 sec
RG 137.36
DW 13.300 usec
DE 20.43 usec
TE 298.0 K
F2 - Processing parameters
SI 65536
SF 150.9028085 MHz
WDW EM
SSB 0
LB 1.00 Hz
GB 0
PC 1.40
165.44
147.98
146.53
142.80
127.54
122.00
115.64
113.33
55.94
53.08
190 180 170 160 150 140 130 120 110 100 90 80 70 60 50 40 30 20 ppm

1f

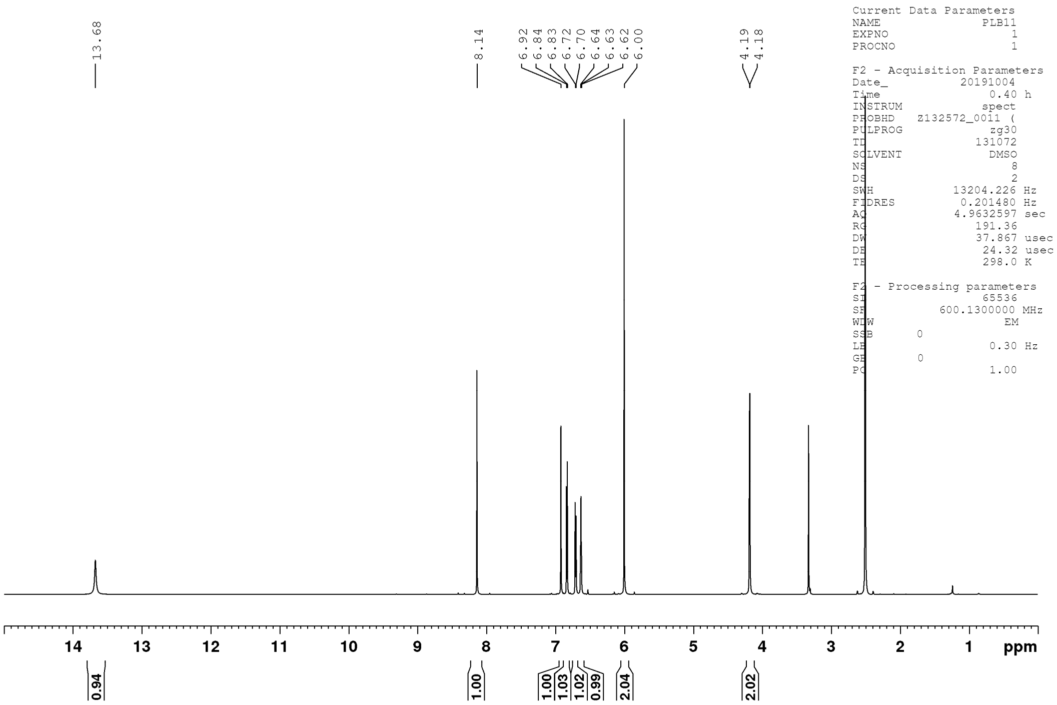
13.68
8.14
6.92
6.84
6.83
6.72
6.70
6.64
6.63
6.62
6.00
4.19
4.18
Current Data Parameters
NAME PLB11
EXPNO 1
PROCNO 1
F2 - Acquisition Parameters
Date_ 20191004
Time 0.40 h
INSTRUM spect
PROBHD Z132572_0011 (
PULPROG zg30
TD 131072
SOLVENT DMSO
NS 8
DS 2
SWH 13204.226 Hz
FIDRES 0.201480 Hz
AQ 4.9632597 sec
RG 191.36
DW 37.867 usec
DE 24.32 usec
TE 298.0 K
F2 - Processing parameters
SI 65536
SF 600.1300000 MHz
WDW EM
SSB 0
LB 0.30 Hz
GB 0
PC 1.00
14 13 12 11 10 9 8 7 6 5 4 3 2 1 ppm
0.94
1.00
1.00
1.03
1.02
0.99
2.04
2.02

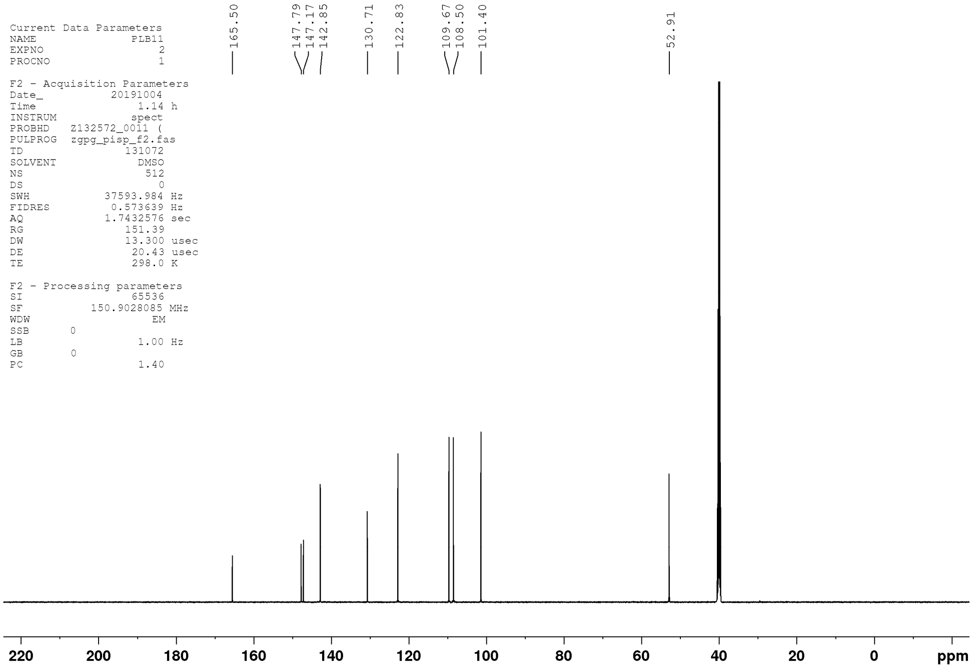
Current Data Parameters
NAME PLB11
EXPNO 2
PROCNO 1
F2 - Acquisition Parameters
Date_ 20191004
Time 1.14 h
INSTRUM spect
PROBHD Z132572_0011 (
PULPROG zgpg_pisp_f2.fas
TD 131072
SOLVENT DMSO
NS 512
DS 0
SWH 37593.984 Hz
FIDRES 0.573639 Hz
AQ 1.7432576 sec
RG 151.39
DW 13.300 usec
DE 20.43 usec
TE 298.0 K
F2 - Processing parameters
SI 65536
SF 150.9028085 MHz
WDW EM
SSB 0
LB 1.00 Hz
GB 0
PC 1.40
165.50
147.79
147.17
142.85
130.71
122.83
109.67
108.50
101.40
52.91
220 200 180 160 140 120 100 80 60 40 20 0 ppm

1g

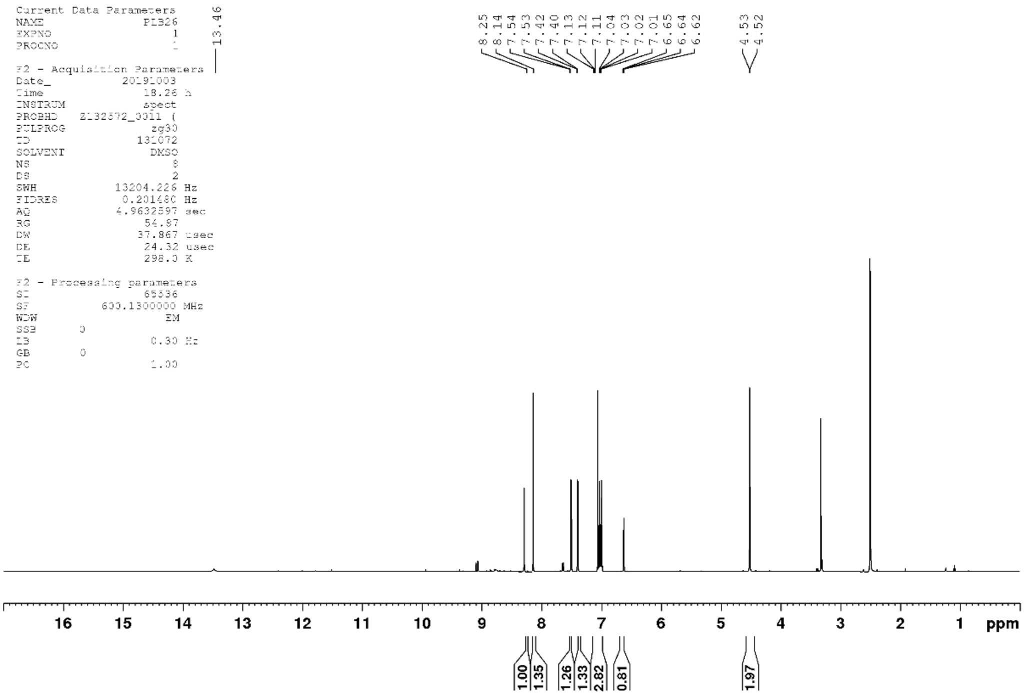

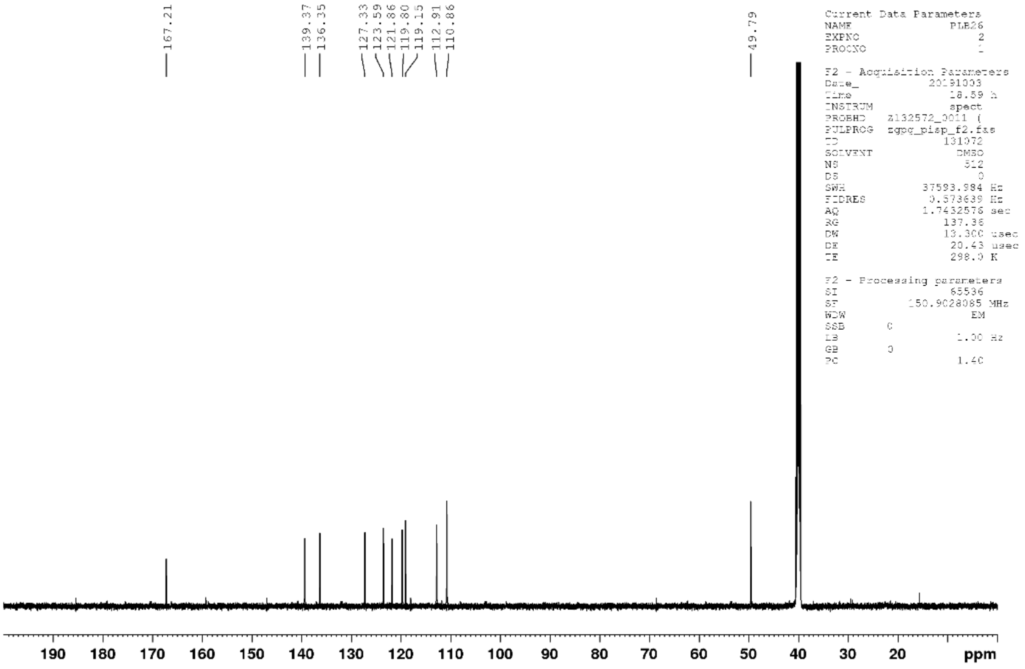

2a

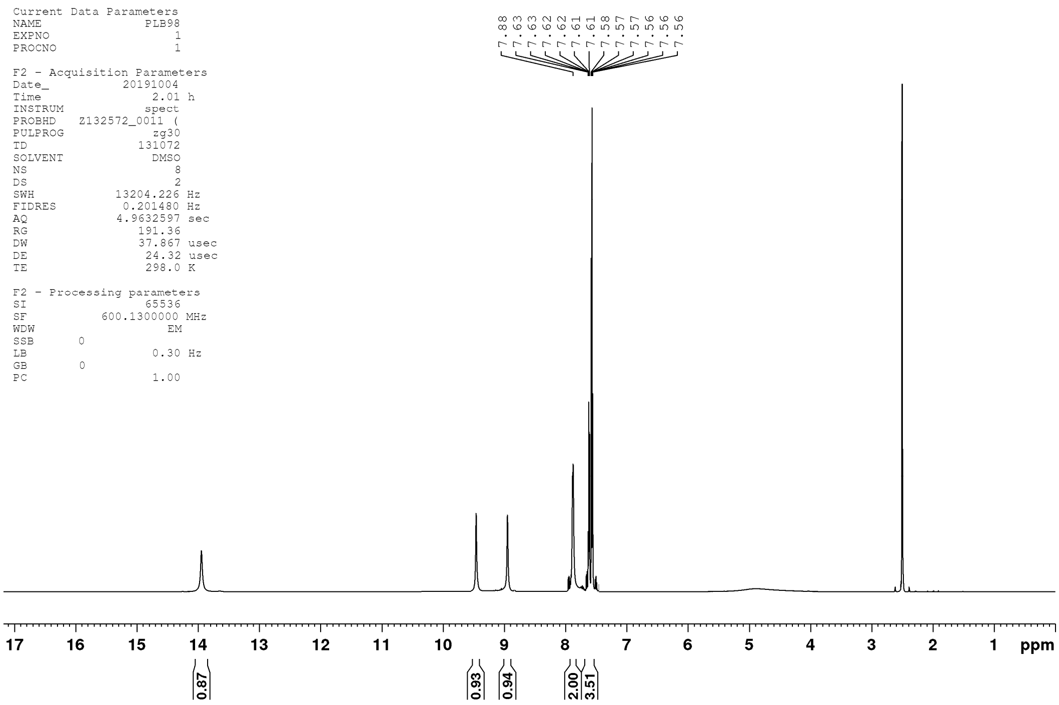
Current Data Parameters
NAME PLB98
EXPNO 1
PROCNO 1
F2 - Acquisition Parameters
Date_ 20191004
Time 2.01 h
INSTRUM spect
PROBHD Z132572_0011 (
PULPROG zg30
TD 131072
SOLVENT DMSO
NS 8
DS 2
SWH 13204.226 Hz
FIDRES 0.201480 Hz
AQ 4.9632597 sec
RG 191.36
DW 37.867 usec
DE 24.32 usec
TE 298.0 K
F2 - Processing parameters
SI 65536
SF 600.1300000 MHz
WDW EM
SSB 0
LB 0.30 Hz
GB 0
PC 1.00
7.88
7.63
7.63
7.62
7.62
7.61
7.61
7.58
7.57
7.57
7.56
7.56
7.56
17 16 15 14 13 12 11 10 9 8 7 6 5 4 3 2 1 ppm
0.87
0.93
0.94
2.00
3.51

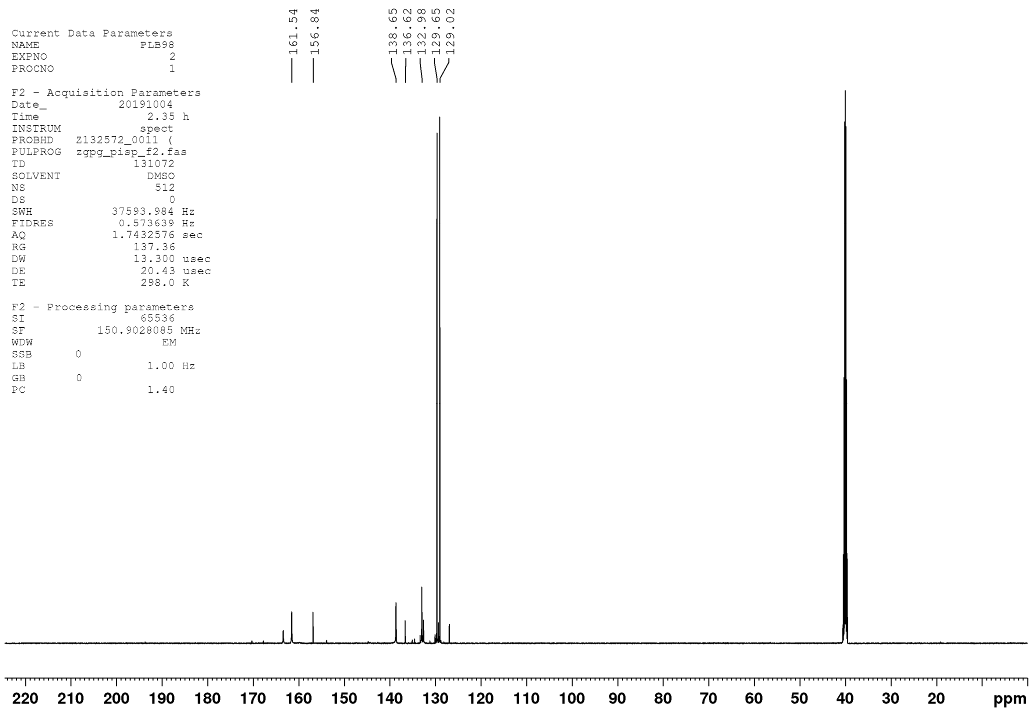
Current Data Parameters
NAME PLB98
EXPNO 2
PROCNO 1
F2 - Acquisition Parameters
Date_ 20191004
Time 2.35 h
INSTRUM spect
PROBHD Z132572_0011 (
PULPROG zgpg_pisp_f2.fas
TD 131072
SOLVENT DMSO
NS 512
DS 0
SWH 37593.984 Hz
FIDRES 0.573639 Hz
AQ 1.7432576 sec
RG 137.36
DW 13.300 usec
DE 20.43 usec
TE 298.0 K
F2 - Processing parameters
SI 65536
SF 150.9028085 MHz
WDW EM
SSB 0
LB 1.00 Hz
GB 0
PC 1.40
161.54
156.84
138.65
136.62
132.98
129.65
129.02
220 210 200 190 180 170 160 150 140 130 120 110 100 90 80 70 60 50 40 30 20 ppm

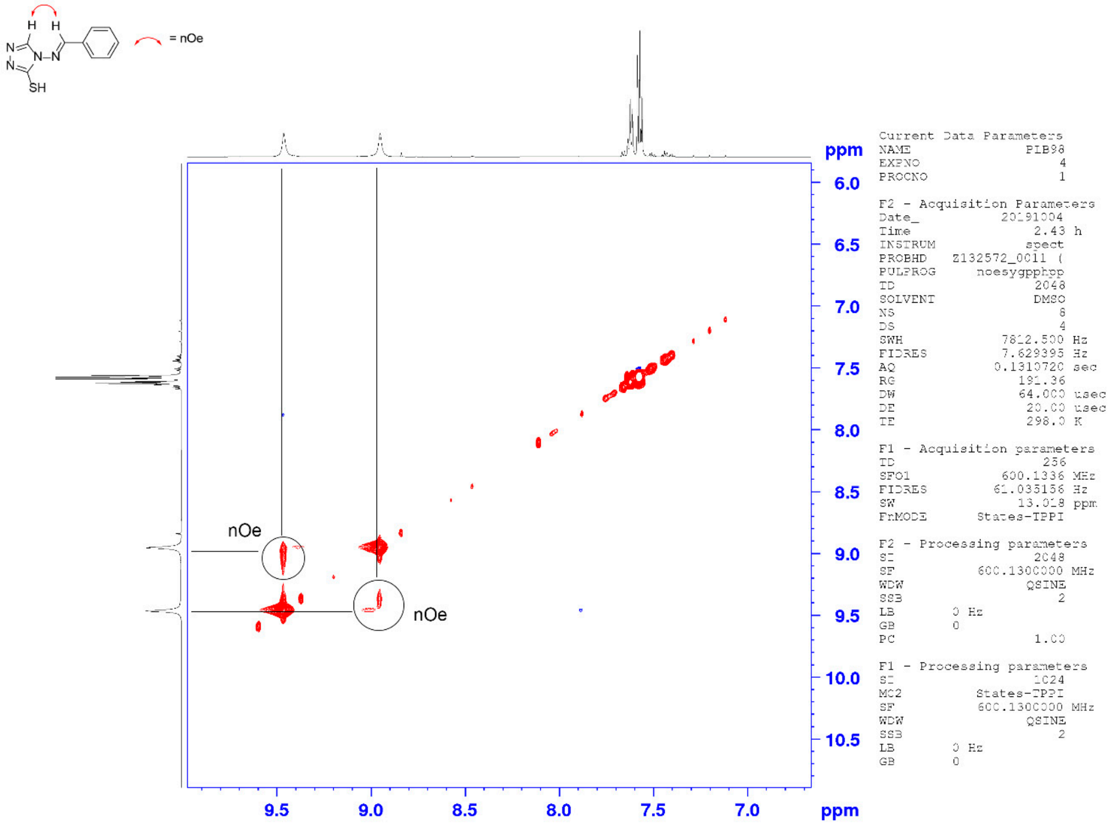

= nOe
nOe
nOe
ppm
6.0
6.5
7.0
7.5
8.0
8.5
9.0
9.5
10.0
10.5
9.5
9.0
8.5
8.0
7.5
7.0
ppm
Current Data Parameters
NAME PIB98
EXPNO 4
PROCNO 1
F2 - Acquisition Parameters
Date_ 20191004
Time 2.43 h
INSTRUM spect
PROBHD Z132572_0011 (
PULPROG noesygpphpp
TD 2048
SOLVENT DMSO
NS 8
DS 4
SWH 7812.500 Hz
FIDRES 7.629395 Hz
AQ 0.1310720 sec
RG 191.36
DW 64.000 usec
DE 20.00 usec
TE 298.0 K
F1 - Acquisition parameters
TD 256
SFO1 600.1336 MHz
FIDRES 61.035156 Hz
SW 13.018 ppm
FnMODE States-TPPI
F2 - Processing parameters
SI 2048
SF 600.1300000 MHz
WDW QSINE
SSB 2
LB 0 Hz
GB 0
PC 1.00
F1 - Processing parameters
SI 1024
MC2 States-TPPI
SF 600.1300000 MHz
WDW QSINE
SSB 2
LB 0 Hz
GB 0

2b

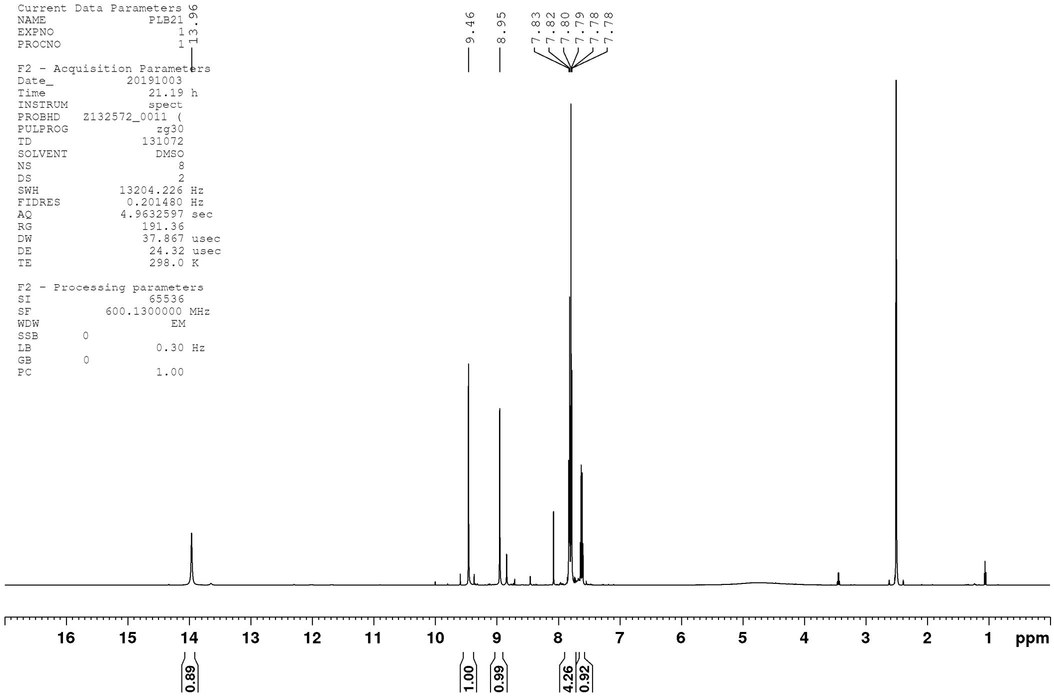
Current Data Parameters
NAME PLB21
EXPNO 1
PROCNO 1
F2 - Acquisition Parameters
Date_ 20191003
Time 21.19 h
INSTRUM spect
PROBHD Z132572_0011 (
PULPROG zg30
TD 131072
SOLVENT DMSO
NS 8
DS 2
SWH 13204.226 Hz
FIDRES 0.201480 Hz
AQ 4.9632597 sec
RG 191.36
DW 37.867 usec
DE 24.32 usec
TE 298.0 K
F2 - Processing parameters
SI 65536
SF 600.1300000 MHz
WDW EM
SSB 0
LB 0.30 Hz
GB 0
PC 1.00
13.96
9.46
8.95
7.83
7.82
7.80
7.79
7.78
7.78
16 15 14 13 12 11 10 9 8 7 6 5 4 3 2 1 ppm
0.89
1.00
0.99
4.26
0.92

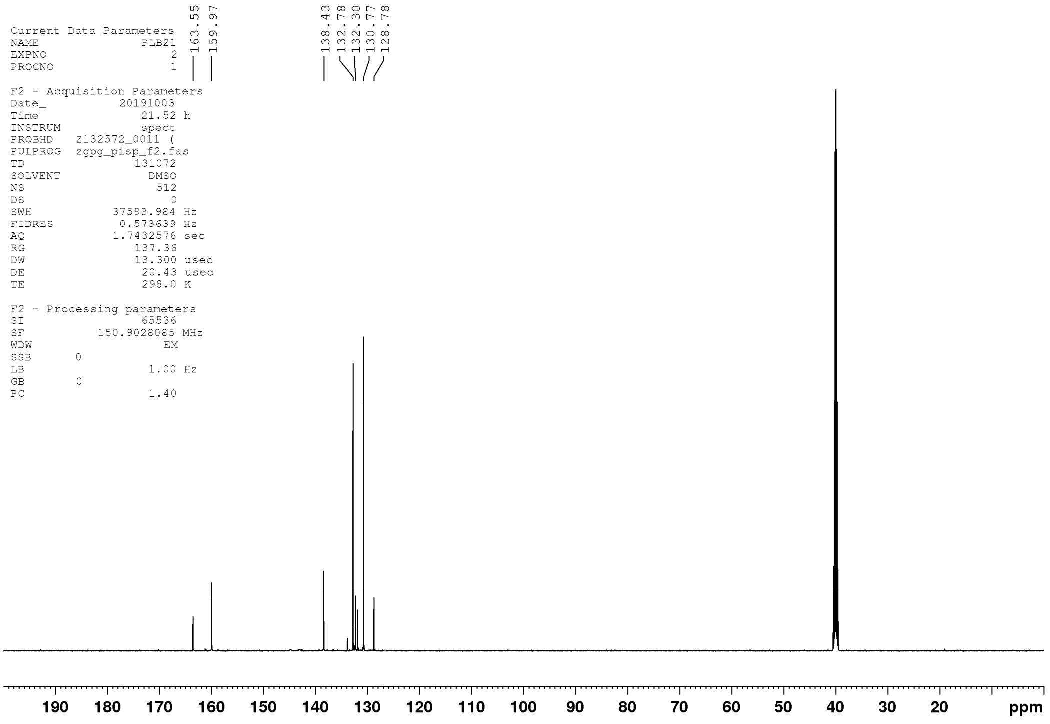
Current Data Parameters
NAME PLB21
EXPNO 2
PROCNO 1
F2 - Acquisition Parameters
Date_ 20191003
Time 21.52 h
INSTRUM spect
PROBHD Z132572_0011 (
PULPROG zgpg_pisp_f2.fas
TD 131072
SOLVENT DMSO
NS 512
DS 0
SWH 37593.984 Hz
FIDRES 0.573639 Hz
AQ 1.7432576 sec
RG 137.36
DW 13.300 usec
DE 20.43 usec
TE 298.0 K
F2 - Processing parameters
SI 65536
SF 150.9028085 MHz
WDW EM
SSB 0
LB 1.00 Hz
GB 0
PC 1.40
163.55
159.97
138.43
132.78
132.30
130.77
128.78
190 180 170 160 150 140 130 120 110 100 90 80 70 60 50 40 30 20 ppm

2c

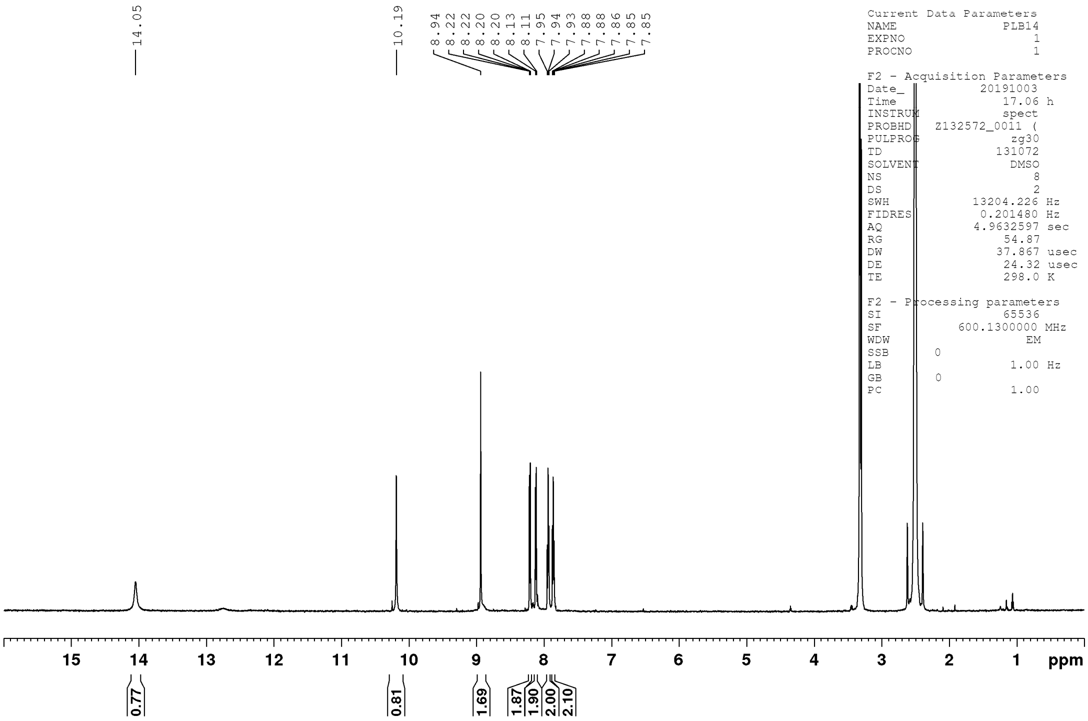

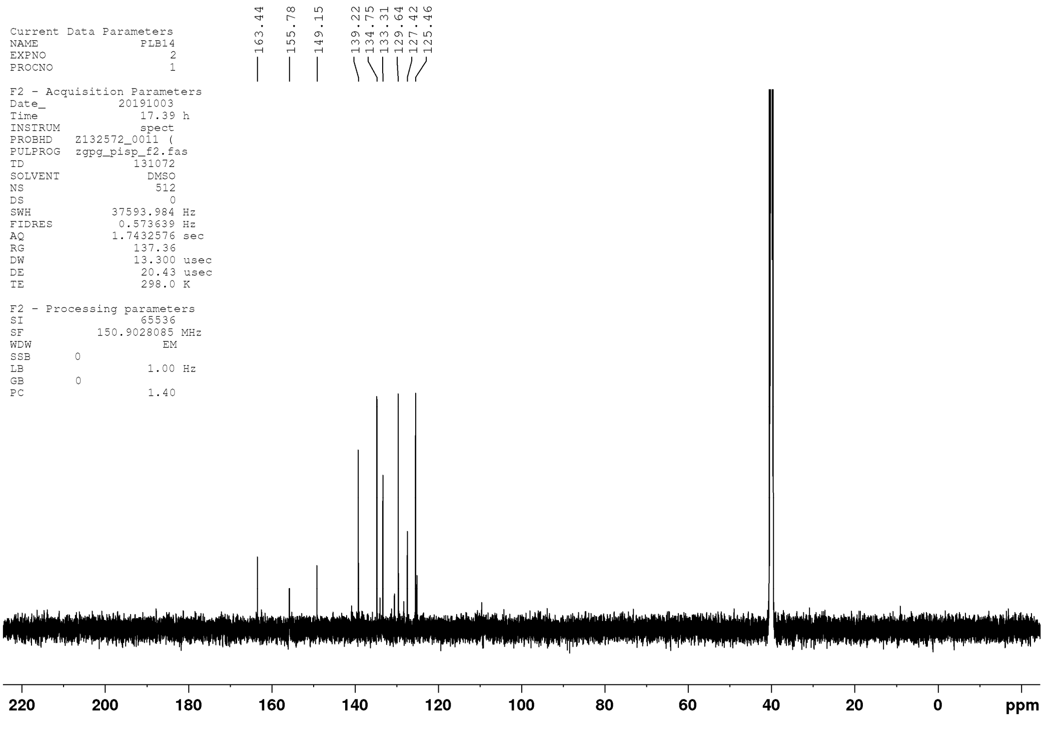

2d

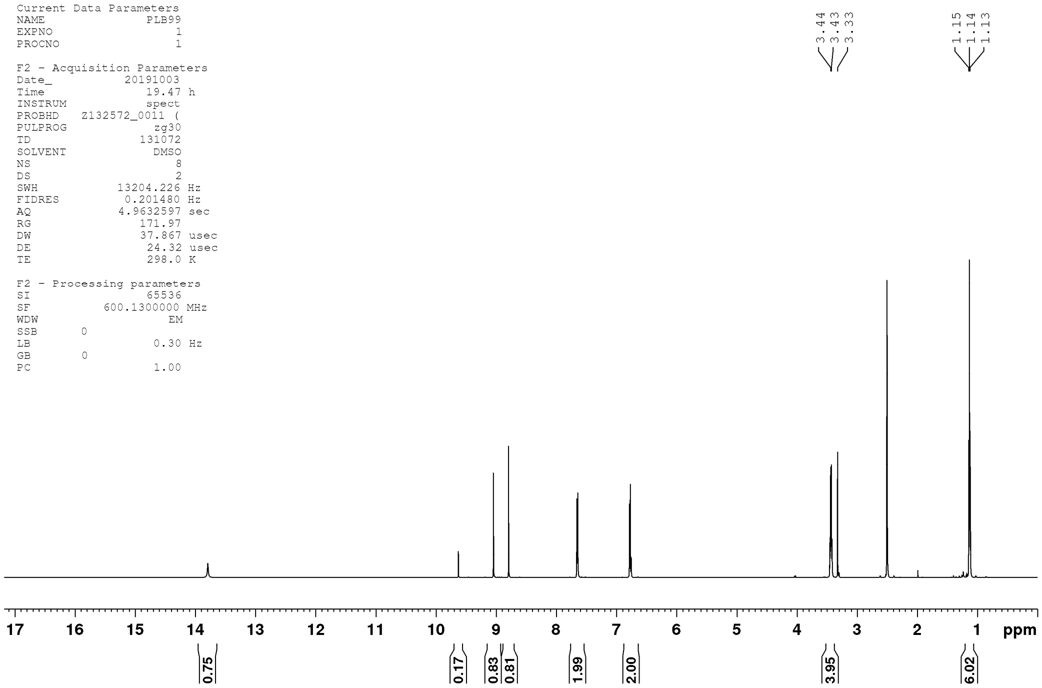
Current Data Parameters
NAME PLB99
EXPNO 1
PROCNO 1
F2 - Acquisition Parameters
Date_ 20191003
Time 19.47 h
INSTRUM spect
PROBHD Z132572_0011 (
PULPROG zg30
TD 131072
SOLVENT DMSO
NS 8
DS 2
SWH 13204.226 Hz
FIDRES 0.201480 Hz
AQ 4.9632597 sec
RG 171.97
DW 37.867 usec
DE 24.32 usec
TE 298.0 K
F2 - Processing parameters
SI 65536
SF 600.1300000 MHz
WDW EM
SSB 0
LB 0.30 Hz
GB 0
PC 1.00
3.44
3.43
3.33
1.15
1.14
1.13
17 16 15 14 13 12 11 10 9 8 7 6 5 4 3 2 1 ppm
0.75
0.17
0.83
0.81
1.99
2.00
3.95
6.02

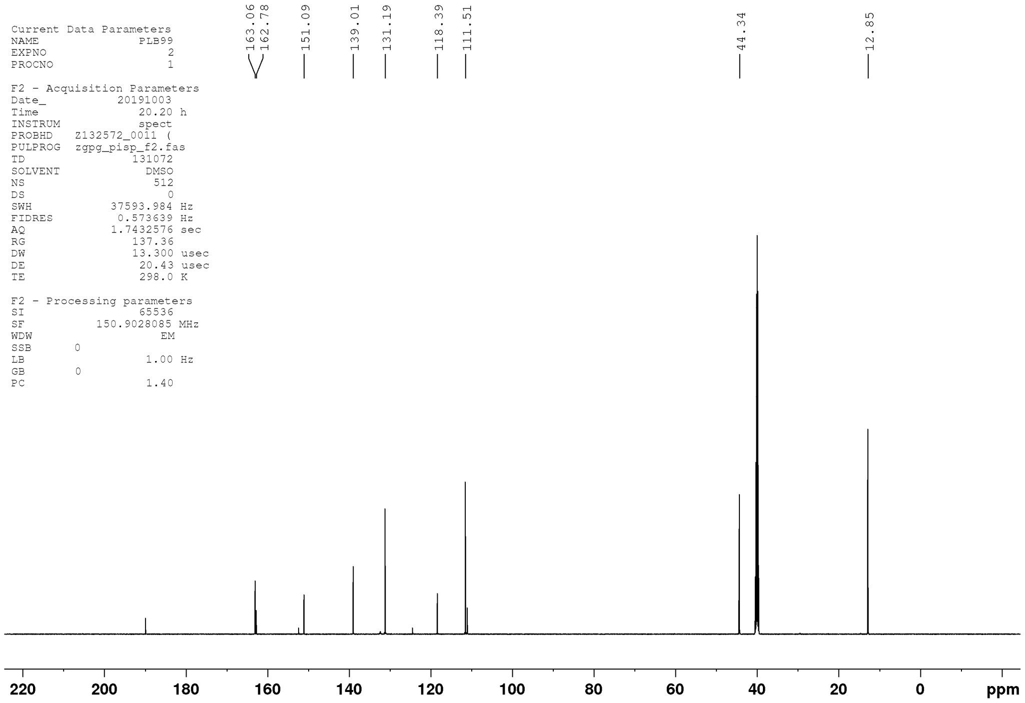
Current Data Parameters
NAME PLB99
EXPNO 2
PROCNO 1
F2 - Acquisition Parameters
Date_ 20191003
Time 20.20 h
INSTRUM spect
PROBHD Z132572_0011 (
PULPROG zgpg_pisp_f2.fas
TD 131072
SOLVENT DMSO
NS 512
DS 0
SWH 37593.984 Hz
FIDRES 0.573639 Hz
AQ 1.7432576 sec
RG 137.36
DW 13.300 usec
DE 20.43 usec
TE 298.0 K
F2 - Processing parameters
SI 65536
SF 150.9028085 MHz
WDW EM
SSB 0
LB 1.00 Hz
GB 0
PC 1.40
163.06
162.78
151.09
139.01
131.19
118.39
111.51
44.34
12.85
220 200 180 160 140 120 100 80 60 40 20 0 ppm

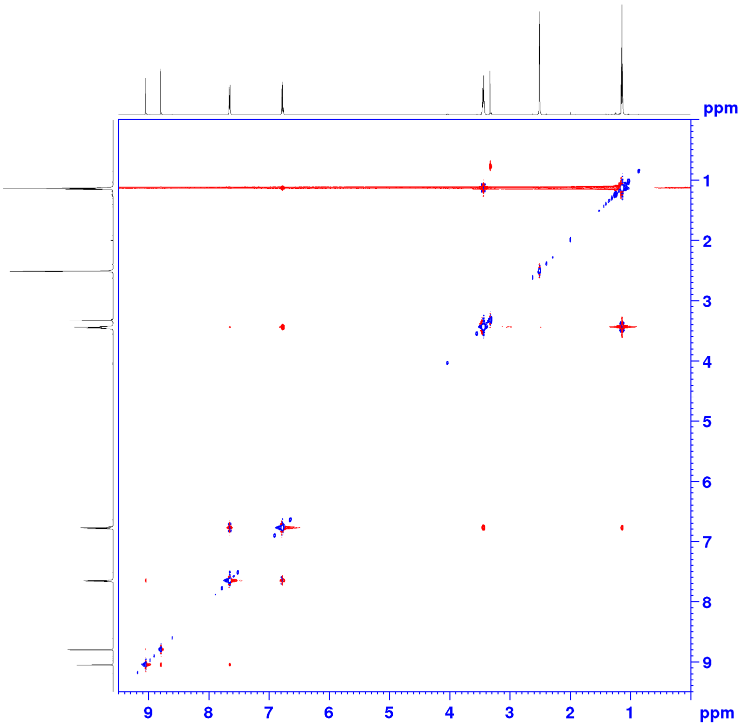


```
Current Data Parameters
NAME              PLB99
EXPNO                 3
PROCNO                1

F2 - Acquisition Parameters
Date_          20191003
Time              20.21 h
INSTRUM           spect
PROBHD   Z132572_0011 (
PULPROG     noesygpphpp
TD                 2048
SOLVENT            DMSO
NS                    8
DS                    4
SWH            7812.500 Hz
FIDRES         7.629395 Hz
AQ            0.1310720 sec
RG               191.36
DW               64.000 usec
DE                20.00 usec
TE                298.0 K

F1 - Acquisition parameters
TD                  256
SFO1           600.1336 MHz
FIDRES        61.035156 Hz
SW               13.018 ppm
FnMODE      States-TPPI

F2 - Processing parameters
SI                 2048
SF          600.1300000 MHz
WDW               QSINE
SSB                   2
LB       0 Hz
GB       0
PC                 1.00

F1 - Processing parameters
SI                 1024
MC2         States-TPPI
SF          600.1300000 MHz
WDW               QSINE
SSB                   2
LB       0 Hz
GB       0
```

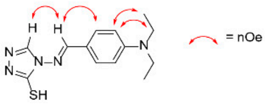

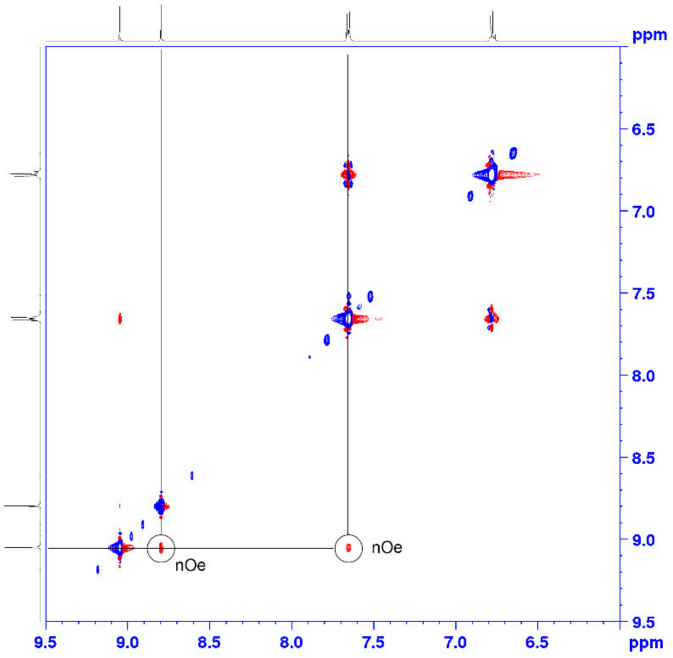


```
Current Data Parameters
NAME              PLB99
EXPNO                 3
PROCNO                1

F2 - Acquisition Parameters
Date_          20191003
Time              20.21 h
INSTRUM           spect
PROBHD   Z132572_0011 (
PULPROG     noesygpphpp
TD                 2048
SOLVENT            DMSO
NS                    8
DS                    4
SWH            7812.500 Hz
FIDRES         7.629395 Hz
AQ            0.1310720 sec
RG               191.36
DW               64.000 usec
DE                20.00 usec
TE                298.0 K

F1 - Acquisition parameters
TD                  256
SFO1           600.1336 MHz
FIDRES        61.035156 Hz
SW               13.018 ppm
FnMODE      States-TPPI

F2 - Processing parameters
SI                 2048
SF          600.1300000 MHz
WDW               QSINE
SSB                   2
LB       0 Hz
GB       0
PC                 1.00

F1 - Processing parameters
SI                 1024
MC2         States-TPPI
SF          600.1300000 MHz
WDW               QSINE
SSB                   2
LB       0 Hz
GB       0
```

2e

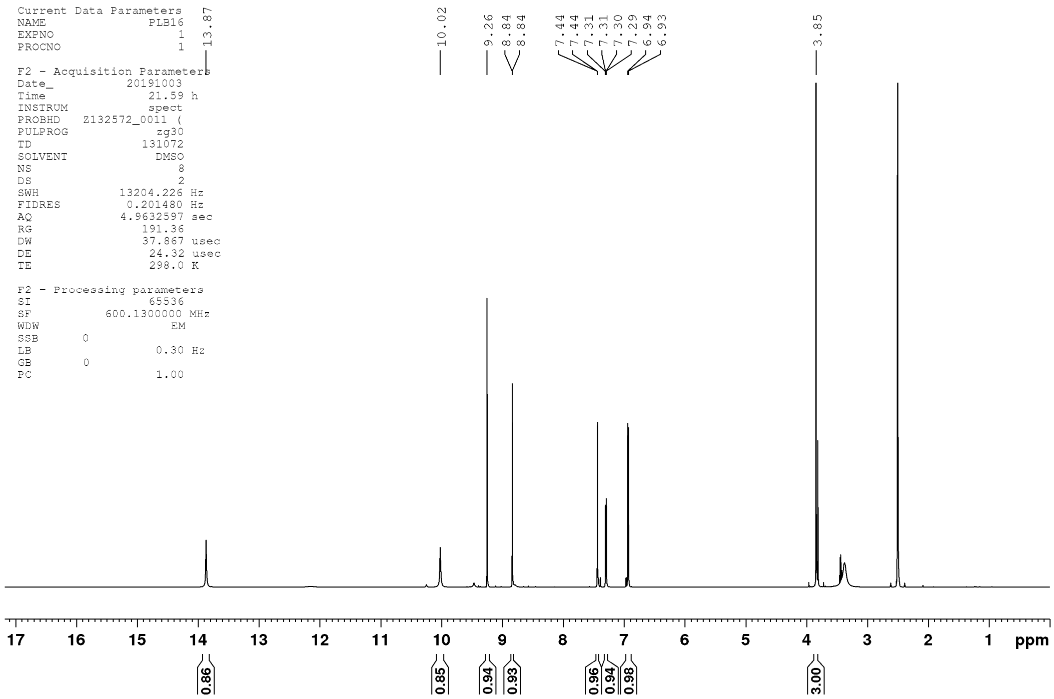
Current Data Parameters
NAME PLB16
EXPNO 1
PROCNO 1
F2 - Acquisition Parameters
Date_ 20191003
Time 21.59 h
INSTRUM spect
PROBHD Z132572_0011 (
PULPROG zg30
TD 131072
SOLVENT DMSO
NS 8
DS 2
SWH 13204.226 Hz
FIDRES 0.201480 Hz
AQ 4.9632597 sec
RG 191.36
DW 37.867 usec
DE 24.32 usec
TE 298.0 K
F2 - Processing parameters
SI 65536
SF 600.1300000 MHz
WDW EM
SSB 0
LB 0.30 Hz
GB 0
PC 1.00
13.87
10.02
9.26
8.84
8.84
7.44
7.44
7.31
7.31
7.30
7.29
6.94
6.93
3.85
17 16 15 14 13 12 11 10 9 8 7 6 5 4 3 2 1 ppm
0.86
0.85
0.94
0.93
0.96
0.94
0.98
3.00

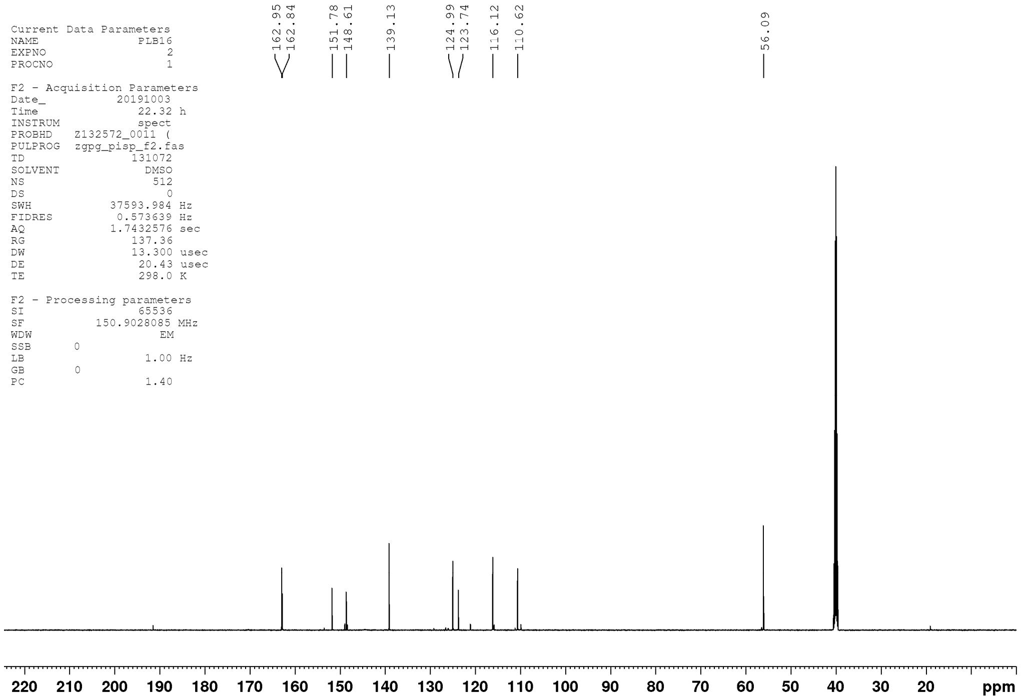
Current Data Parameters
NAME PLB16
EXPNO 2
PROCNO 1
F2 - Acquisition Parameters
Date_ 20191003
Time 22.32 h
INSTRUM spect
PROBHD Z132572_0011 (
PULPROG zgpg_pisp_f2.fas
TD 131072
SOLVENT DMSO
NS 512
DS 0
SWH 37593.984 Hz
FIDRES 0.573639 Hz
AQ 1.7432576 sec
RG 137.36
DW 13.300 usec
DE 20.43 usec
TE 298.0 K
F2 - Processing parameters
SI 65536
SF 150.9028085 MHz
WDW EM
SSB 0
LB 1.00 Hz
GB 0
PC 1.40
162.95
162.84
151.78
148.61
139.13
124.99
123.74
116.12
110.62
56.09
220 210 200 190 180 170 160 150 140 130 120 110 100 90 80 70 60 50 40 30 20 ppm

2f

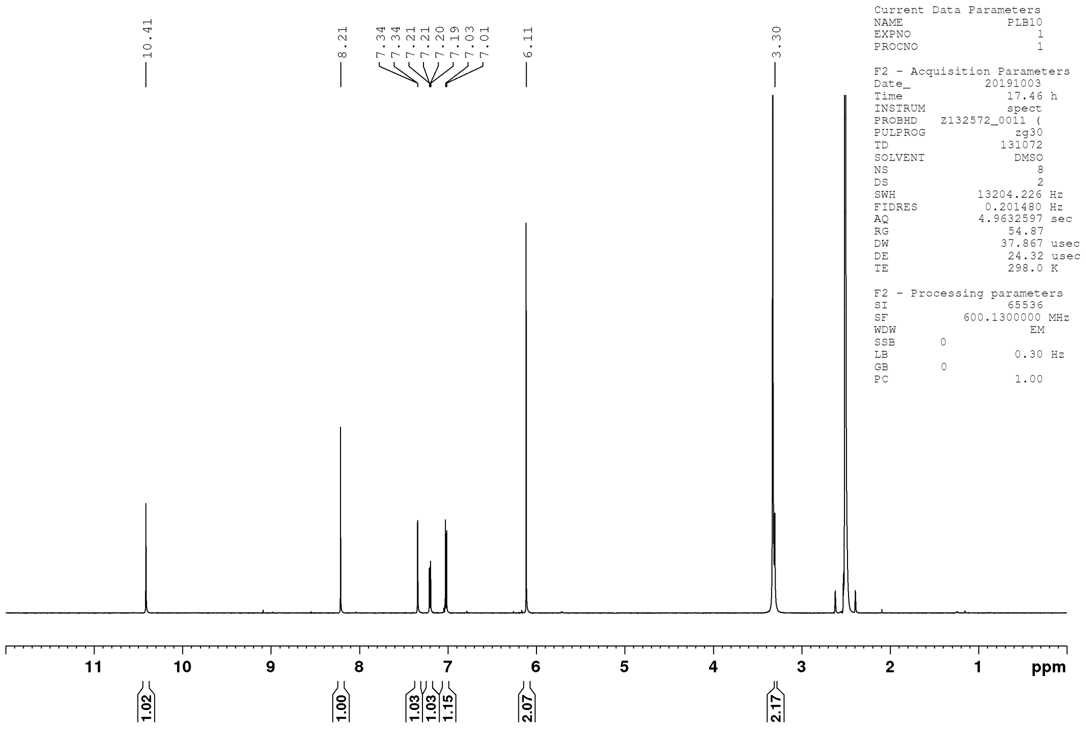
10.41
8.21
7.34
7.34
7.21
7.21
7.20
7.19
7.03
7.01
6.11
3.30
Current Data Parameters
NAME PLB10
EXPNO 1
PROCNO 1
F2 - Acquisition Parameters
Date_ 20191003
Time 17.46 h
INSTRUM spect
PROBHD Z132572_0011 (
PULPROG zg30
TD 131072
SOLVENT DMSO
NS 8
DS 2
SWH 13204.226 Hz
FIDRES 0.201480 Hz
AQ 4.9632597 sec
RG 54.87
DW 37.867 usec
DE 24.32 usec
TE 298.0 K
F2 - Processing parameters
SI 65536
SF 600.1300000 MHz
WDW EM
SSB 0
LB 0.30 Hz
GB 0
PC 1.00
11 10 9 8 7 6 5 4 3 2 1 ppm
1.02
1.00
1.03
1.03
1.15
2.07
2.17

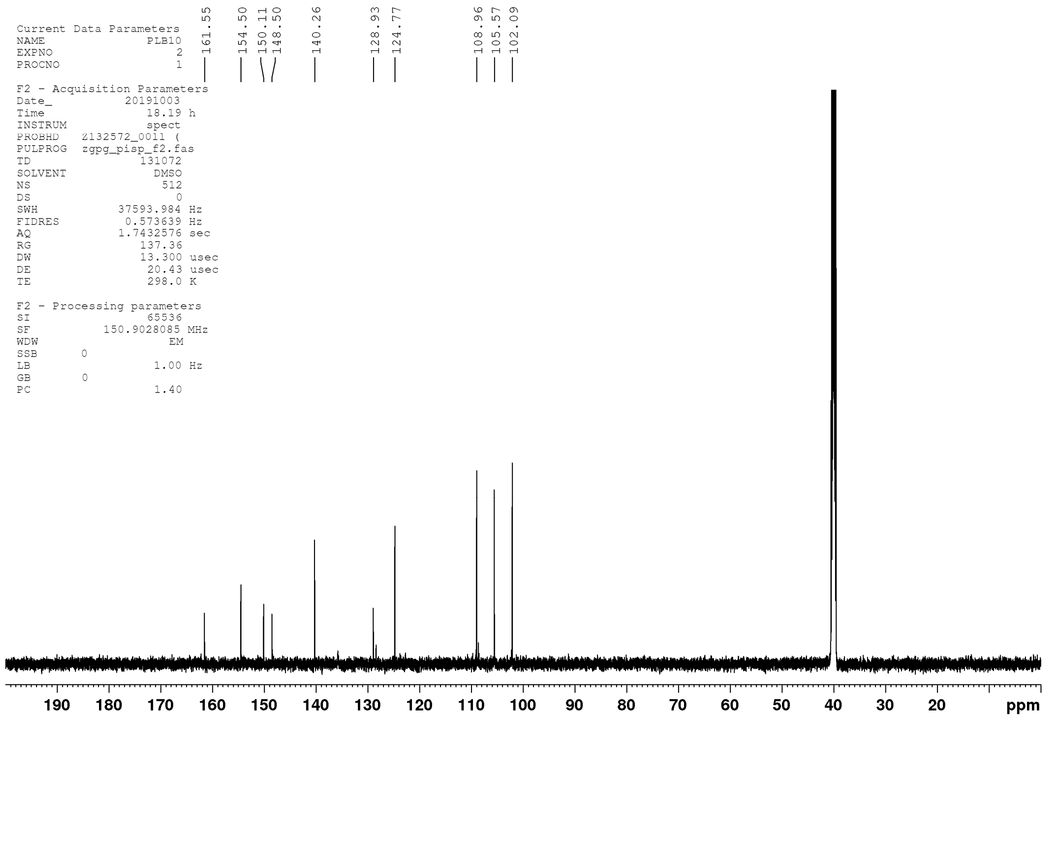
Current Data Parameters
NAME PLB10
EXPNO 2
PROCNO 1
F2 - Acquisition Parameters
Date_ 20191003
Time 18.19 h
INSTRUM spect
PROBHD Z132572_0011 (
PULPROG zgpg_pisp_f2.fas
TD 131072
SOLVENT DMSO
NS 512
DS 0
SWH 37593.984 Hz
FIDRES 0.573639 Hz
AQ 1.7432576 sec
RG 137.36
DW 13.300 usec
DE 20.43 usec
TE 298.0 K
F2 - Processing parameters
SI 65536
SF 150.9028085 MHz
WDW EM
SSB 0
LB 1.00 Hz
GB 0
PC 1.40
161.55
154.50
150.11
148.50
140.26
128.93
124.77
108.96
105.57
102.09
190 180 170 160 150 140 130 120 110 100 90 80 70 60 50 40 30 20 ppm

2g

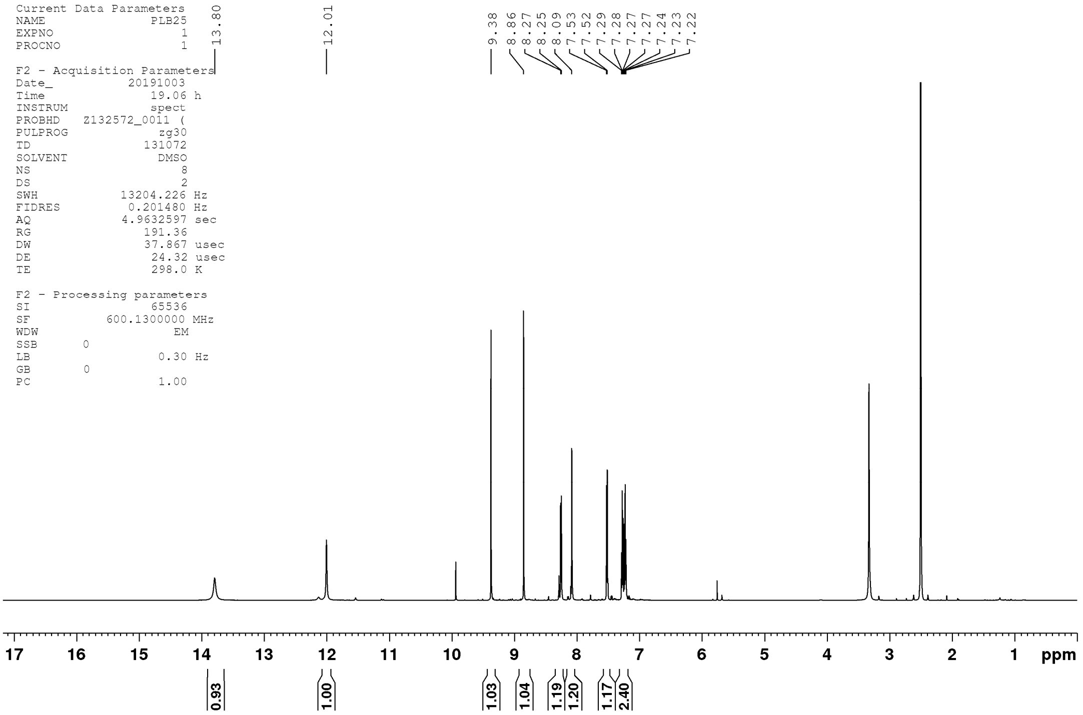

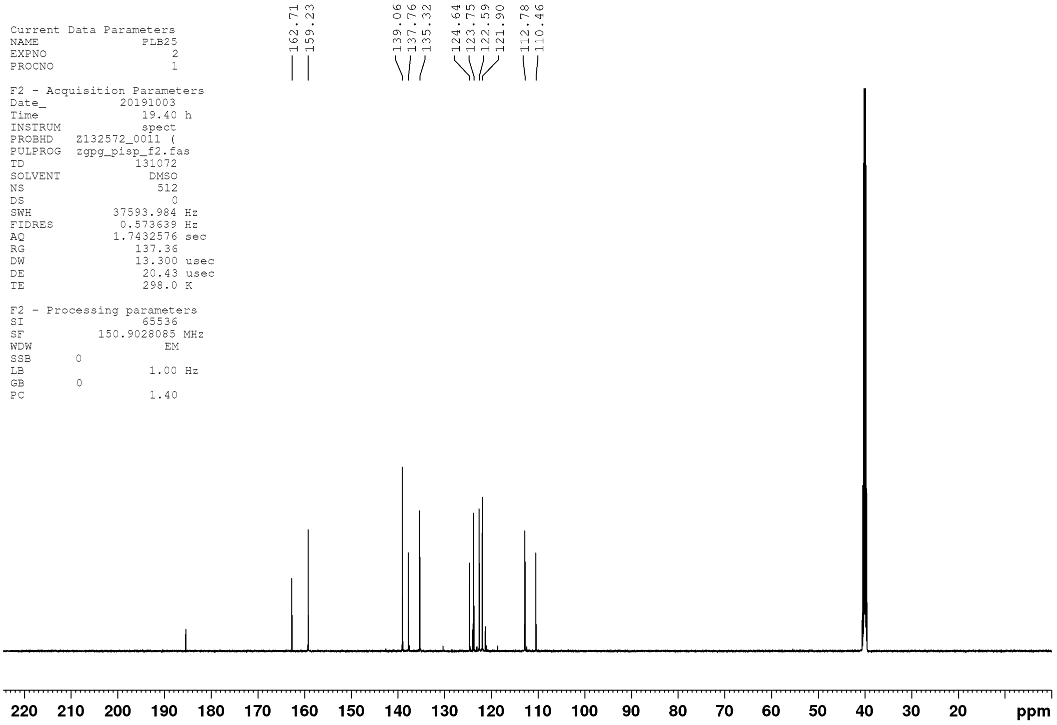

Supplement: Supplementary file 1 [file pharmaceuticals-13-00052-s001.pdf]
